# Fingerprinting Acute Digestive Diseases by Untargeted NMR Based Metabolomics

**DOI:** 10.3390/ijms19113288

**Published:** 2018-10-23

**Authors:** Panteleimon G. Takis, Antonio Taddei, Riccardo Pini, Stefano Grifoni, Francesca Tarantini, Paolo Bechi, Claudio Luchinat

**Affiliations:** 1 Giotto Biotech, S.r.l, Via Madonna del Piano 6, 50019 Sesto Fiorentino, Italy; takis@giottobiotech.com; 2 Department of Surgery and Translational Medicine, School of Medicine, Careggi University Hospital, Largo Brambilla 3, 50134 Florence, Italy; antonio.taddei@unifi.it (A.T.); paolo.bechi@unifi.it (P.B.); 3 Department of Experimental and Clinical Medicine, University of Florence, Largo Brambilla 3, 50134 Florence, Italy; riccardo.pini@unifi.it (R.P.); francesca.tarantini@unifi.it (F.T.); 4 Department of Emergency Medicine and Surgery, Careggi University Hospital, 50134 Florence, Italy; grifonis@aou-careggi.toscana.it; 5 Magnetic Resonance Center (CERM), University of Florence, Via L. Sacconi 6, 50019 Sesto Fiorentino, Italy

**Keywords:** acute digestive disease, biliary colic, pancreatitis, metabolomics, intestinal ischemia, ileus, intestinal strangulated obstruction

## Abstract

Precision medicine may significantly contribute to rapid disease diagnosis and targeted therapy, but relies on the availability of detailed, subject specific, clinical information. Proton nuclear magnetic resonance (^1^H–NMR) spectroscopy of body fluids can extract individual metabolic fingerprints. Herein, we studied 64 patients admitted to the Florence main hospital emergency room with severe abdominal pain. A blood sample was drawn from each patient at admission, and the corresponding sera underwent ^1^H–NMR metabolomics fingerprinting. Unsupervised Principal Component Analysis (PCA) analysis showed a significant discrimination between a group of patients with symptoms of upper abdominal pain and a second group consisting of patients with diffuse abdominal/intestinal pain. Prompted by this observation, supervised statistical analysis (Orthogonal Partial Least Squares–Discriminant Analysis (OPLS-DA)) showed a very good discrimination (>90%) between the two groups of symptoms. This is a surprising finding, given that neither of the two symptoms points directly to a specific disease among those studied here. Actually herein, upper abdominal pain may result from either symptomatic gallstones, cholecystitis, or pancreatitis, while diffuse abdominal/intestinal pain may result from either intestinal ischemia, strangulated obstruction, or mechanical obstruction. Although limited by the small number of samples from each of these six conditions, discrimination of these diseases was attempted. In the first symptom group, >70% discrimination accuracy was obtained among symptomatic gallstones, pancreatitis, and cholecystitis, while for the second symptom group >85% classification accuracy was obtained for intestinal ischemia, strangulated obstruction, and mechanical obstruction. No single metabolite stands up as a possible biomarker for any of these diseases, while the contribution of the whole ^1^H–NMR serum fingerprint seems to be a promising candidate, to be confirmed on larger cohorts, as a first-line discriminator for these diseases.

## 1. Introduction

In emergency rooms, especially in the case of acute symptoms, the central dogma of patients’ treatment effectiveness consists of rapid disease diagnosis and subsequent targeted cure. Personalized medicine is progressively acquiring importance for both of these purposes and for drug development [1,2]. In turn, a deeper knowledge of each individual’s metabolome is becoming important for defining individual phenotypes. In recent years, analysis of the human metabolome through the systematic study of bio-specimens (biofluids, tissues, etc.) has strongly developed [3].

The human metabolome consists of several thousands of small molecules (150–2000 Da), which are present in all body fluids in a very wide range of concentrations (from nanomolar to molar) [4,5]. Blood contains hundreds of molecules—from lipids, such as phospholipids, fatty acids, steroids to amino acids, proteins, glucose, etc.—that are trafficked to and from our organs and tissues. Among body fluids, the whole ensemble of the small molecules circulating in blood, usually called the blood metabolic fingerprint, bears a clear reflection of the physiological condition of the organism. The metabolic fingerprint can be extracted by various spectroscopic and/or spectrometric techniques such as ^1^H–NMR spectroscopy, gas chromatography–mass spectrometry (GC–MS), and liquid chromatography–mass spectrometry (LC–MS) [6].

So far, a few metabolomics studies have addressed several digestive diseases, including inflammatory bowel disease (IBD) [7,8] and liver and bile tract diseases [9], reporting the presence of partially specific biomarkers in serum, urine and fecal extracts that could help early and successful diagnosis.

Herein, we focus upon two groups of three different acute gastrointestinal diseases. The main characteristic of the two groups is that the symptoms of the patients are initially expressed by a severe abdominal pain along with nausea, etc. The first group encompasses bile tract diseases, namely, symptomatic gallstones, cholecystitis, and pancreatitis, and the second one comprises obstructive bowel diseases, namely, intestinal ischemia, intestinal strangulated obstruction, and intestinal mechanical obstruction cases.

The symptoms of the three diseases in the first group mostly consist of severe upper abdominal pain, sometimes associated with vomit, and may require immediate hospitalization and treatment. However, treatment of the three conditions is quite different [10,11]. The three diseases of the second group may also have similar symptoms, consisting of diffuse abdominal pain with abdominal swelling and symptoms of bowel obstruction. Although all of them require urgent surgery, treatment is, again, different. All the aforementioned diseases represent not infrequent causes of access to emergency room. For all of them an early diagnosis is mandatory, and sometimes critical for survival [12,13,14,15]. It should be noted that the two groups of diseases could be quite easily diagnosed by experienced medical doctors since the abdominal pain is expressed in different regions of the abdomen along with other symptoms, however, several blood and physical exams should be performed before the initial diagnosis and so far, there have been no evidence for these diseases discrimination by untargeted metabolomics via serum profiling.

The present study aims at assessing whether serum NMR-based metabolomics fingerprints for these diseases can be obtained and can help diagnosis in patients admitted to the emergency room with acute abdominal pain. Namely, we tried to test if the serum metabolic profile, extracted by NMR, could discriminate and “map” the two groups of acute gastrointestinal diseases, while we attempted to decompose each group into its diseases and get some preliminary evidence of their fingerprint in serum NMR profiles.

## 2. Results

### 2.1. Diseases Discrimination According to Their Symptoms

**Figure 1:**
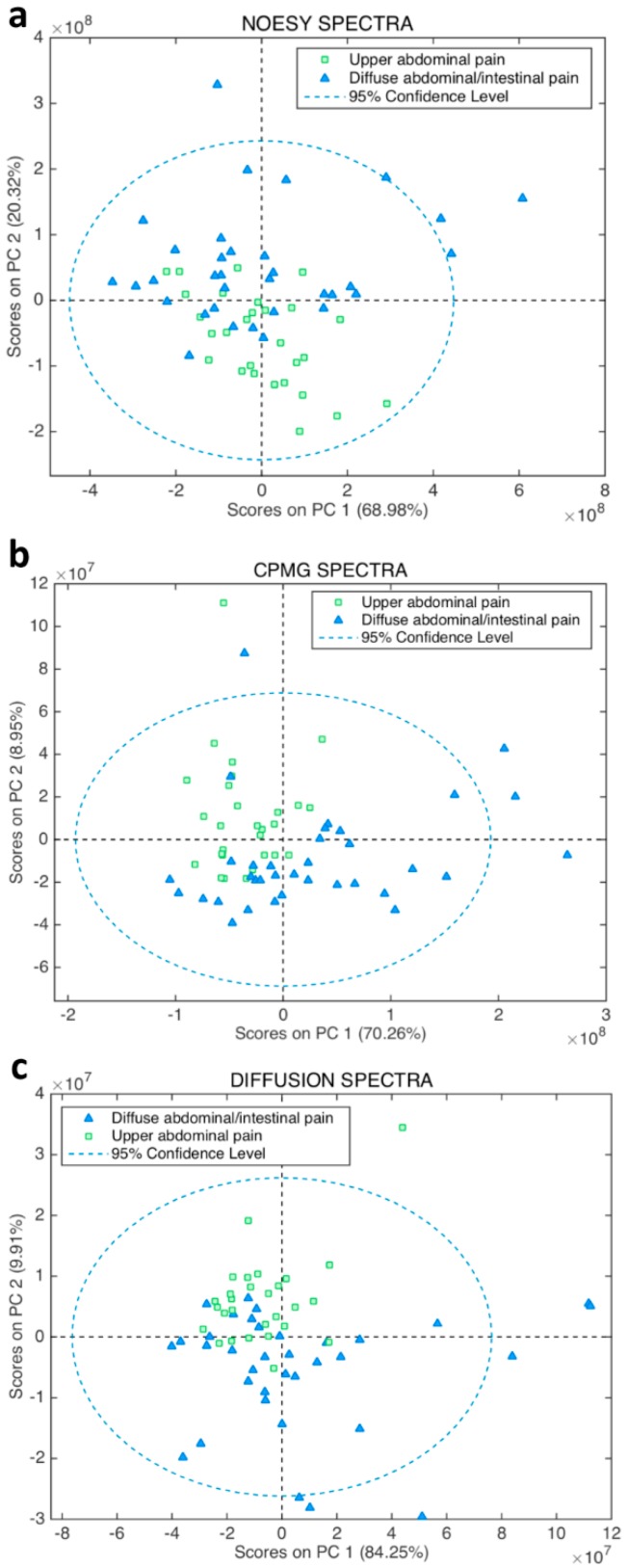
2D Score plot of the Principal Component Analysis (PCA) analysis for the NMR serum spectra of the patients participated in this study. The data are colored according to two groups: diseases with sever upper abdominal pain (*n* = 29, green squares) and diffuse abdominal/intestinal pain (*n* = 35, light blue triangles). PCA analyses were performed for the serum (**a**) Nuclear Overhauser Effect Spectroscopy (NOESY), (**b**) Carr-Purcell-Meiboom-Gill (CPMG), (**c**) diffusion NMR spectra.

**Figure 2:**
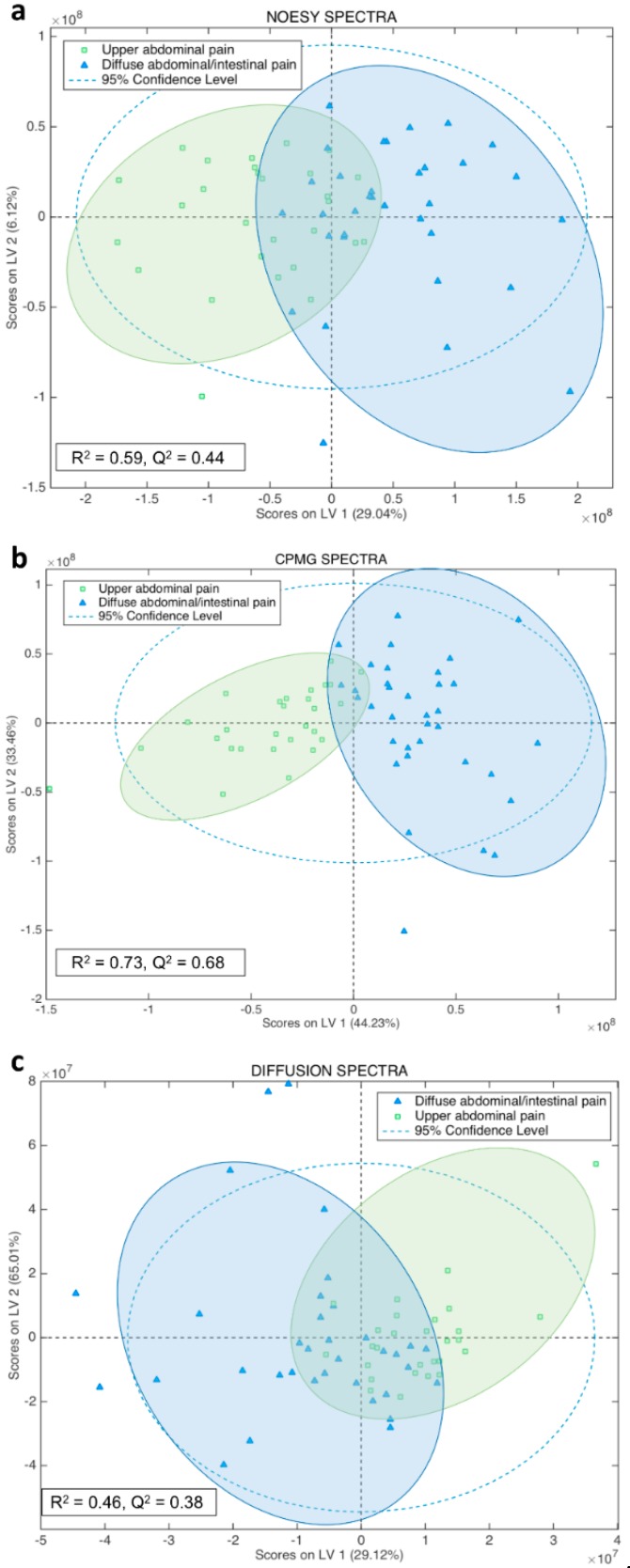
2D score plots of the Orthogonal Partial Least Squares–Discriminant Analysis (OPLS-DA) classification of two groups of diseases according to their symptomatology, namely one group of digestive diseases causing sever upper abdominal pain (bile tract diseases) and one group of diseases expressed with diffuse abdominal/intestinal pain (bowel obstructive diseases). The OPLS-DA models are based upon (**a**) NOESY, (**b**) CPMG and (**c**) diffusion NMR spectra, respectively. The second latent variable (LV) is the orthogonal component. In addition, the R^2^, Q^2^ values and the 95% confidence ellipse of each group are depicted.

**Figure 3:**
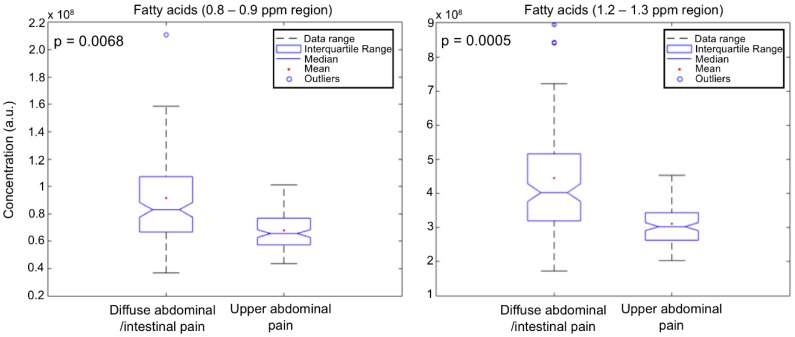
Boxplots and calculated probability values (*p*) from Kruskal–Wallis non-parametric analysis of variance test of the most weighted variables (metabolites’ NMR signals) for the two group of diseases classification. Boxplots depict the concentration (in arbitrary units, a.u.) of some of the weighted metabolites for the groups classification. The analysis was performed after integration of the NMR signals of the weighted metabolites from the serum CPMG spectra.

**Figure 4:**
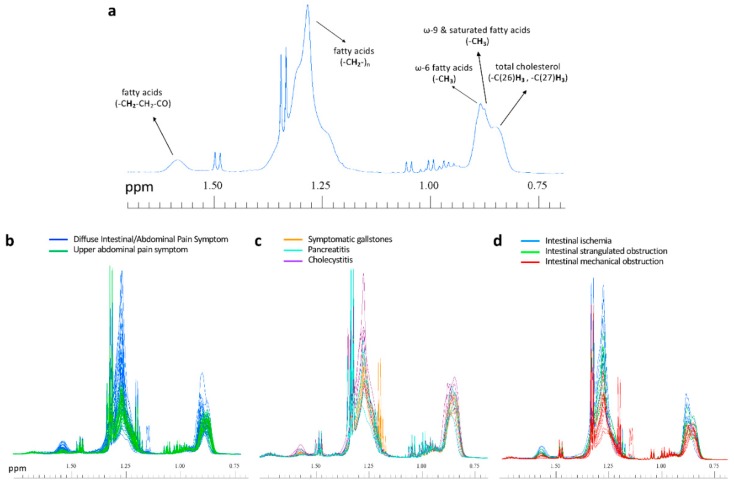
(**a**) NMR spectral region of a serum sample, focusing on the signals of aliphatic protons from fatty acids and total cholesterol (see Section 4.3 of the manuscript for the assignment details). (**b**–**d**) All spectra employed for the studied groups of symptoms and diseases, colored accordingly and focused on the NMR regions of fatty acids and total cholesterol.

Unsupervised multivariate analysis by Principal Component Analysis (PCA) of all types of serum spectra (Figure 1) prompted a good discrimination of the diseases that focus on the upper abdominal pain from those that exhibit intestinal and diffuse abdominal pain. Orthogonal Partial Least Squares–Discriminant Analysis (OPLS-DA) supervised analysis of all type of spectra (Figure 2) showed that the Carr-Purcell-Meiboom-Gill (CPMG) spectra produce the highest classification accuracy (Figure 2b, Table 1). In particular, CPMG spectra profile is able to discriminate with >90% accuracy the two major groups of the diseases. Permutation tests (500 random iterations) were performed in order to identify any over-fit of both cross validated and not CPMG model (Figure S1). A probability table was derived with the calculated probabilities (*p* values) from pairwise Wilcoxon signed test, pairwise signed test as well as randomization *t*-test (Table S1). *p* values indicated that the corresponding models were significantly different at the 95% confidence limit and unlikely to be random. The loading plot of the OPLS-DA analysis (Figure S2) from the CPMG spectra hinted to some significant NMR signals that contribute to the discrimination. These signals correspond to an alteration of fatty acids concentration, which appears to be elevated in the case of the intestinal diseases compared to the upper abdomen diseases (Figure 3). In particular, a thorough inspection of the CPMG spectra (Figure 4a) shows that ω-6, ω-9 and unsaturated fatty acids are probably increased (Figure 4b), along with a potential elevation of total cholesterol levels (Figure 4b). Further, detailed assignment of specific fatty acids is hindered from their signal overlap as well as the presence of lipoproteins. It should be noted that not only fatty acids contribute to the discrimination, since the calculated discrimination accuracy of the diseases is <65% when only these spectral regions are considered. This indicates that the whole serum NMR profile of the CPMG spectra leads to the high classification accuracy, acting as a “collective” biomarker [16].

### 2.2. Biliary/Pancreatic Diseases

PCA analysis of the serum NOESY spectra (Figure S3) from patients affected by biliary colic due to symptomatic gallstones, cholecystitis, and pancreatitis suggested a probable discrimination among these diseases. In fact, the subsequent OPLS-DA supervised analysis resulted in a good classification of the three diseases (Figures S4a and S5a). In particular, the NMR fingerprints can be clustered in three groups, which exhibit high discrimination accuracy, as assessed by the cross-validated confusion matrix (Table S2). In particular for the cholecystitis, cross-validated results showed a very high discrimination with accuracy >90%.

**Figure 5:**
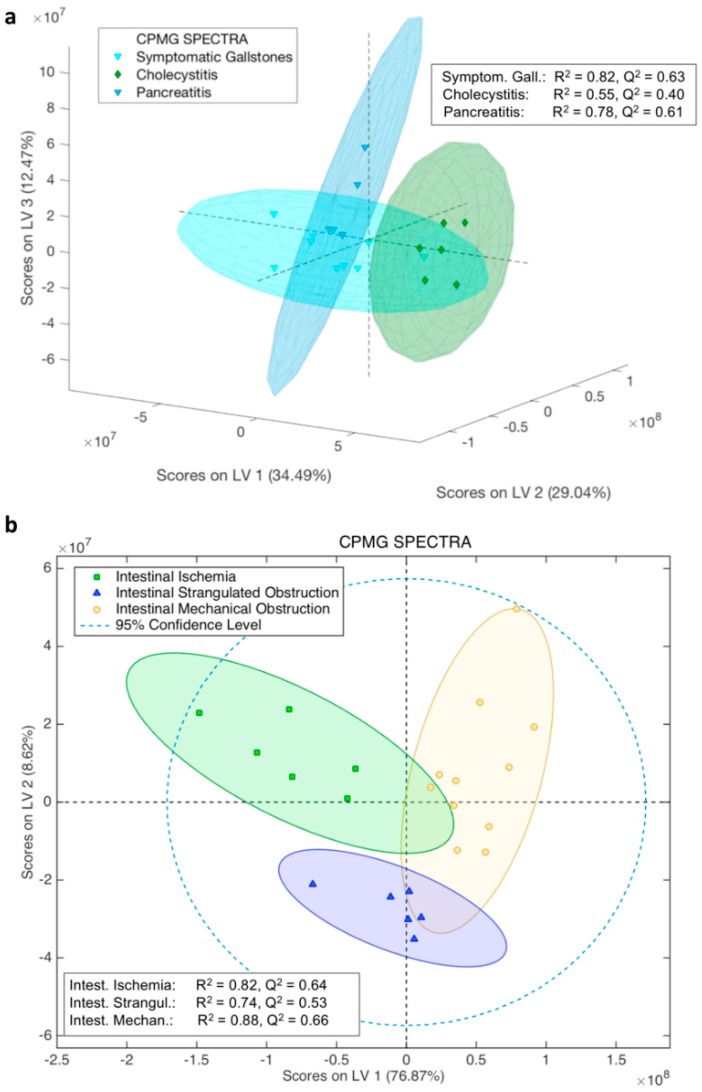
3D and 2D score plots of the OPLS-DA classification of: (**a**) symptomatic gallstones, cholecystitis, and pancreatitis; (**b**) of intestinal ischemia, intestinal strangulated obstruction, and intestinal mechanical obstruction cases, respectively, along with R^2^ and Q^2^ values. OPLS-DA models are derived from serum ^1^H-NMR CPMG spectra. The 3D score plot has been constructed after the analysis of the serum spectra and the three first latent variables (LVs) components were used (the third one is the orthogonal component), which were also used for the final model production, since after the third component the mean classification error in the model was increasing (Figure S5b). Extra viewing angles of the 3D score plot from the CPMG spectra model are depicted in Figure S7. Except for the three groups samples distribution, the 95% confidence ellipse of each group is depicted.

Following the same supervised statistical approach, this time employing the CPMG (Figure S6) serum spectra, the accuracy results of the derived model were higher (Figure 5a and Figure S7, and Table 2). In particular, CPMG spectra seem to be the best predictors for biliary colic and pancreatitis, with accuracy >90% (Table 2). This finding was further supported by trying to classify each disease against the other two (Figure S8). From these supervised statistical analyses (Figure S8), it is immediately appreciated that each one of the biliary/pancreatic diseases is very well classified against the other two, with accuracy >85%.

Despite the low number of samples used for the construction of the models, we also attempted to detect any statistically significant difference in the concentration of individual metabolites among the serum profiles of the three diseases. Thus, we employed the LVs weighted variables (i.e., responsible for the disease discrimination) of the statistical analysis based on either NOESY (Figure S9a) or CPMG (Figure S9b) spectra. Figures S10 and S11 focus on the weighted variables of the loading plots (i.e., metabolite signals that contribute the most to the classification based on the variable importance in projection (VIP) scores for each group) for the NOESY and CPMG models, respectively. It appears that, in both cases, statistically significant differences are mainly (but not only, since the whole NMR profile contributes to the discrimination) determined by ω-6, ω-9 and unsaturated fatty acids (Figures S12a, S13a and Figure 4c), glycoproteins, and phospholipids. Despite the limited number of samples, the univariate statistical analyses showed that fatty acids and phospholipids concentrations may be indeed higher in the case of biliary colic due to symptomatic gallstones, whereas patients diagnosed with cholecystitis exhibit lower concentrations (Figure 4c, Figures S12a and S13a).

It should be mentioned that the analysis of the diffusion spectra (Figures S14 and S15, and Table S3), which mostly contain ^1^H-NMR signals of macromolecules, did not provide higher accuracy than NOESY and CPMG spectra. This is another proof that the whole NMR fingerprint of NOESY and CPMG spectra leads to the discrimination of the bile tract diseases and not only fatty acids, glycoproteins, and phospholipids signals.

In order to test the predicting ability of the NOESY and CPMG models, we employed the extra serum samples (nine), which were not used for the model construction (see section of Material and Methods, paragraph: patients’ recruitment for the study). Among them, three corresponded to symptomatic gallstones, three to cholecystitis and three to pancreatitis according to clinical diagnosis. In detail, their NMR spectra (both NOESY and CPMG) were used as test datasets (see Figure S6) in order to predict through our models, the disease of each patient and comparison of the results with the medical diagnosis. The prediction results by the CPMG spectra (Figure S16a, Table S4) could be considered more than satisfying given the small number of data used to build our models, since eight out of nine patients were correctly assigned. The predicting ability of the NOESY model was slightly lower (Table S4 and Figure S16b) than the CPMG, as expected from their cross-validated results (Table 2 and Table S2).

### 2.3. Intestinal Diseases

By following the same statistical approach as for biliary/pancreatic diseases, NOESY, CPMG, and diffusion spectra were employed for the discrimination of the intestinal diseases, namely intestinal ischemia, strangulated obstruction, and mechanical obstruction cases. The score plots from the multivariate analyses (MVDA) analysis of the three types of spectra are shown in Figure S17a,b, Figure 5b and Figure S18 respectively. In particular, all analyses exhibit high accuracy (Table S5 and Table 3), with the following order of NMR type of spectra from the highest to lowest performance: CPMG > NOESY > DIFFUSION. The accuracy of the CPMG model was further confirmed by testing the classification of each one of the diseases compared to the other two. OPLS-DA analyses showed that each disease is discriminated with at least 76% accuracy against the other two (Figure S19).

Subsequently, following the same procedure as above, we tried to detect the most discriminating metabolites (if any) for each disease. The loading plots (Figure S20) and the VIP scores (Figures S21–S23) indicate some significant metabolite concentration differences for each disease. In particular, the same classes of fatty acids as previously mentioned appear again as a group of metabolites that contribute to the three diseases classification along with total cholesterol. They tend to be highest in intestinal ischemia and lowest in intestinal mechanical obstruction (Figures S12b, S13b and Figure 4d).

Phospholipids (PL) are statistically significant only for the CPMG spectra (Figure S13b), being highest in intestinal strangulated obstruction and lowest in intestinal ischemia cases, respectively.

Predicting ability tests were also performed for all three models (as for the biliary/pancreatic diseases), by means of three extra serum samples of intestinal ischemia, two of intestinal strangulated obstruction, and seven of intestinal mechanical obstruction. The CPMG model exhibits the highest predicting ability, since all the test data of both intestinal ischemia and intestinal strangulated obstruction were correctly assigned, and five out of seven intestinal mechanical obstruction test samples were also accurately predicted (Table S6 and Figure S24), in agreement with the clinical diagnosis. Overall, 10 out of 12 patients were successfully diagnosed from the CPMG spectra. As a whole, our the CPMG data shows good diagnostic ability, especially for the more severe cases, i.e., intestinal ischemia and intestinal strangulated obstruction.

## 3. Discussion

The main finding of the present study is represented by the possibility of identifying NMR-based metabolomics maps, which could help the early differential diagnosis of some acute digestive diseases. To test this possibility, two different groups of acute digestive diseases for which a differential diagnosis is required, were considered (biliary/pancreatic diseases for the first group, and intestinal diseases for the second group). It should be noted that each of the two groups of diseases could be diagnosed by experienced medical doctors since the abdominal pain is expressed in different regions of the abdomen along with other symptoms, however, several blood and physical exams should be performed before the initial diagnosis.

Our results showed for the first time that NMR based untargeted metabolomics could discriminate with very high accuracy these two groups of diseases (>90%) and facilitate the initial diagnosis of medical doctors. The statistical analysis of the two groups of diseases showed that probably fatty acids are in higher concentration in the patients with intestinal obstructive diseases compared to the patients with biliary problems. Although fatty acids concentration in blood varies a lot, depending on dietary habits, etc., our result could be supported by the fact that in a lot of intestinal inflammatory diseases, fatty acids binding protein levels seem to increase [17]. This could also justify an increase of fatty acids concentration in blood stream [17].

On the contrary, it is more difficult to further discriminate, based only on clinical symptoms, the three subcases in each group of the above-mentioned diseases. The first group of digestive diseases consists in biliary/pancreatic diseases which usually require a number of clinical as well as laboratory data [10,11,18,19] in order to achieve a correct diagnosis. Abdominal ultrasound is necessary, but CT-scan, MRI, and endoscopic ultrasound may also play important roles for their diagnosis [18,19,20,21]. It is worth mentioning that all these diagnostic tools are time consuming and expensive [22].

Even though there are a small number of samples, our study shows that highest and lowest fatty acid and phospholipid concentrations along with the whole serum NMR profile of small molecular weight metabolites [23] classify symptomatic gallstones and cholecystitis, since the models from the CPMG spectra (better highlighting the sharp NMR signals of small size metabolites) had the best mean predicting ability. In this respect, it is worth mentioning that lipid metabolism has been previously reported as a significant factor for bile tract disease development [24]. It has also been demonstrated that serum polyunsaturated free fatty acids, especially arachidonic acid, were significantly elevated in patients experiencing acute pancreatitis [24]. Moreover, hydrolysis of phospholipid serum content by phospholipase A_2_ leads to polyunsaturated fatty acid production [24]. This finding may also contribute to explain our observation of phospholipids increase in pancreatitis and symptomatic gallstones when compared to cholecystitis (Figures S13a and S14a). In addition, gallbladder bile analysis of gallstone patients indicated very high amounts of serum mannose-containing glycoproteins suggesting that they may play an important role in the pathogenesis of gallstone disease [25]. This hypothesis is consistent with our preliminary data, which show that glycoproteins are elevated in the patients with symptomatic gallstones.

The second group of digestive diseases that were considered in this study consists of intestinal ischemia and intestinal strangulated or mechanical obstructions. All these diseases exhibit high mortality rate, unless they are diagnosed at an early stage. For an early diagnosis, blood biomarkers, such as I-FABP (intestinal fatty acids binding protein) levels [15,26] or D-(−)-lactate [27] have been previously proposed. However, the results do not seem to be specific and indicative for each disease, although serum D-(−)-lactate concentration has been found to be high in some abdominal diseases, and the I-FABP levels seem to increase in many intestinal inflammatory diseases [27] and they have also been reported as early biomarkers for intestinal ischemia [28]. Their increased levels may be due to the key role of I-FABP proteins in regenerating cellular membranes and intestinal tissues by transferring phospholipids and fatty acid degradation products to the damaged areas [26,29,30,31]. According to these reports, our observation of elevated fatty acid concentrations in intestinal ischemia when compared with intestinal mechanical obstruction could be related to increased I-FABP proteins concentration. Moreover, plasma I-FABP was recently shown to be an early biomarker for small bowel strangulated obstruction [28], consistent with our finding of significantly higher fatty acid concentration levels in strangulated obstruction when compared to mechanical intestinal obstruction.

The present encouraging data also point to systems biology approaches, including metabolomics profiling, as significant contributors of biomarkers for various digestive diseases [16,23,32]. Previous studies dealing with metabolomics and specifically NMR based metabolomics showed that the entire NMR profile—or fingerprint—could be used as a collective ‘biomarker’ [9,16,23], which is the case for our study.

## 4. Materials and Methods

This study has been approved by the local Ethical Committee (Prot 2010/0012462–approved 08-April-2010).

### 4.1. Patients Recruitment for the Study

All the patients who reached the emergency room of our hospital for a first admission due to severe abdominal pain were included into the study. At admittance, and 2–6 h later, two blood samples were collected before any other diagnostic exam and before administration of any therapy. Sixty-four patients (30 females and 34 males) were considered for the study. Thirty-five of them exhibited symptoms of diffuse abdominal pain and of bowel obstruction. Their diagnosis revealed in 9 of them intestinal ischemia, in 8 intestinal strangulated obstruction and in 18 intestinal mechanical obstruction. Twenty-nine of the patients came to the emergency with symptoms of sever upper abdominal pain and their diagnosis showed in 12 of them a biliary colic due to symptomatic gallstones, in 8 pancreatitis and in 9 cholecystitis. As expected, an accurate history collection revealed for 21 out of the 64 of patients some additional illnesses (diabetes, cardiovascular diseases, hypertension and Parkinson’s disease).

Initially, we exploited all serum samples of the patients in order to see if NMR untargeted metabolomics could discriminate the group of diseases with symptoms of acute upper abdominal pain from the other group of diseases that focus in the intestinal area with symptoms of diffuse abdominal and intestinal pain.

In the second step of our study, we decided to decompose each group according to its enclosed diseases. To do this, initially, we excluded the patients that had extra illnesses from the multivariate analyses (MVDA), while employing them as an independent test set for validation of each statistical model. As they could possibly show reflection of comorbidities, these additional samples would be even more valuable if the test were to result successful. Overall, the group of each disease was based upon the samples of 6 intestinal ischemia, 6 intestinal strangulated obstructions, 11 intestinal mechanical obstructions, 9 biliary colics due to symptomatic gallstones, 5 pancreatitis and 6 cholecystitis. As above mentioned, the other serum samples we employed as test data for the evaluation of MVDA models’ predictability.

### 4.2. Blood Collection and Preparation

Blood was drawn at admission to the emergency room, after acquiring the appropriate informed consent (explaining to the patients the aims of the study and ensuring them the complete anonymity of the data collected). Each sample consisted of 8–10 cc of peripheral blood and was immediately ultra-centrifuged for about 10–15 min at 1300 RCF. Serum samples were then subdivided in small aliquots (about 500–600 microliters each), and immediately stored at −80 °C.

### 4.3. NMR Spectra Processing and Statistical Analysis Methods

NMR samples preparation and NMR spectra acquisition procedures are reported in detail in the Supplementary Material. All statistical analyses (multivariate–univariate) were performed by using MATLAB (MathWorks, version R2014b). In particular, we employed the PLS_Toolbox version 8.0.2 (2015) (Eigenvector Research, Inc., Manson, WA, USA 98831; software available at http://www.eigenvector.com), built in the MATLAB statistical toolbox, and homemade scripts in the MATLAB environment. Despite the relatively small number of samples, the results of this pilot study turned out to be encouraging, even according to a recent critical analysis of the correct use of statistics [33].

Bucketed NMR data was mean-centered, by subtracting the average value of each X variable so as to simplify the interpretation of the models. Principal Component Analysis (PCA) was initially employed to detect any outliers. For the discrimination of the diseases supervised Orthogonal Partial Least Squares–Discriminant Analysis (OPLS-DA) was used. Numerous studies report and validate (especially for serum/plasma metabolomics studies) the ability of OPLS-DA to maximize the independence of the variables, resulting in better separation of data between/among cohorts, and therefore in better discrimination of individuals suffering from different diseases [34]. Correct and incorrect assignments of each grouped disease were used to define true positives (TP), true negatives (TN), false positives (FP), and false negatives (FN) classification rates and then to estimate the percent sensitivity and specificity, as well as the accuracy of each model. All statistical results, classification and model accuracy/predicting ability indicators were evaluated by the use of cross validation. The over-fitting of the cross-validated and no-cross validated models was examined by permutation tests (500 random iterations) and the derived probabilities (*p* values) less than 0.05 indicate that the models are significantly different at the 95% limit [35,36].Latent variables (LV) plots of the OPLS-DA analysis components and the estimated variable importance in projection (VIP) scores for each disease group (variables with VIP > 1.0 were initially considered statistically significant for each model) were evaluated. In order to detect the weighted variables (i.e., the metabolites’ signals), if any, for the diseases’ discrimination.

After the detection of possible biomarkers, the Kruskal–Wallis non parametric test combined with 2-fold cross-validation [37] was performed for the determination of the statistically significant (*p* < 0.05) variables. Their corresponding boxplots were also produced, depicting their mean, median concentration differences for each disease. Metabolites in the ^1^H-NMR fingerprint were identified with the help of Chenomx NMR suite 8.1 (evaluation license), bibliographic data [38,39], Human Metabolome Database (HMDB) [40] as well as AMIX (Bruker Biospin) metabolites database. The extra validation and predicting ability of our pilot models took place by testing additional serum samples (different from those used for the construction of the models).

### 4.4. Data Deposition

The NMR data have been uploaded in the NIH Common Fund’s Metabolomics Data Repository and Coordinating Center (supported by NIH grant, U01-DK097430) website, the Metabolomics Workbench, http://www.metabolomicsworkbench.org, where it has been assigned Project ID PR000707. The data could be accessed directly via it’s Project DOI: 10.21228/M8911D once the curation phase due its processing is concluded.

## 5. Conclusions

Conclusively, we explored the ability of serum metabolomics by NMR as a tool for discriminating two groups of diseases that their main clinical characteristics are quite different but their initial symptoms are usually expressed with severe pain in the abdominal area. The classification accuracy was very high and the produced models were statistically significant. This finding implies that NMR untargeted metabolomics based upon the whole serum NMR profile could contribute to the initial estimation of diagnosis by the medical doctors.

Furthermore, the “venturesome” decomposition of the two groups of diseases (i.e., six different gastroenterological diseases with few samples for each disease) produced several pilot models that despite the low number of samples showed statistical significance. The OPLS-DA statistical models from CPMG serum spectra of patients experiencing biliary colic, cholecystitis, and pancreatitis, demonstrated the highest accuracy, compared to all other models. Their predicting ability was also tested with extra serum samples, where eight out of nine test samples of biliary colic, pancreatitis, and cholecystitis were accurately predicted. The low number of patients for each disease does not allow for a very significant individual biomarker identification—although some metabolites are significantly different within each disease group—whereas the whole ^1^H–NMR patients’ serum profile already leads to a more robust disease discrimination. The same stands for the group of intestinal diseases, where the models from CPMG serum spectra exhibited accuracy ≥95% and their diagnostic ability was tested on 12 extra serum samples with 10 out of 12 were predicted correctly.

In this study, we provide the first evidence that metabolomics can be predictive for severe gastroenterological diseases with similar symptoms. The results are promising and certainly warrant further studies on larger cohorts to validate the idea that a single, fast, and inexpensive serum NMR profiling could be used as an extra screening tool at the admission to the emergency care.

## Figures and Tables

**Table 1:** Cross-Validation results (confusion matrices) and accuracies from the OPLS-DA analysis of NOESY, CPMG, and diffusion NMR spectra for the diseases with symptoms of severe upper abdominal pain (UAP) vs. the diseases with diffuse abdominal/intestinal pain (DAIP).

	Upper Abdominal Pain Symptom (UAP)	Diffuse Intestinal/Abdominal Pain Symptom (DAIP)
**Predicted/Expressed Symptom**	NOESY SPECTRA (Cross validation)	
Predicted as UAP	25	11
Predicted as DAIP	4	24
Accuracy (%)	77.6	
**Predicted/Expressed Symptom**	CPMG SPECTRA (Cross validation)	
Predicted as UAP	28	3
Predicted as DAIP	1	32
Accuracy (%)	93.8	
**Predicted/Expressed Symptom**	DIFFUSION SPECTRA (Cross validation)	
Predicted as UAP	21	12
Predicted as DAIP	8	23
Accuracy (%)	69.4	

**Table 2:** Cross-Validation results (confusion matrices) and accuracies from the OPLS-DA analysis of the CPMG NMR spectra for symptomatic gallstones, pancreatitis, and cholecystitis.

Predicted/Diagnosed	Symptomatic Gallstones	Cholecystitis	Pancreatitis
	CPMG SPECTRA (Cross validation)		
Predicted as Symptomatic Gallstones	9	0	0
Predicted as Cholecystitis	0	5	1
Predicted as Pancreatitis	0	1	4
Accuracy (%)	100	88.1	90

**Table 3:** Cross-Validation results (confusion matrices) and accuracies from the OPLS-DA analysis of the CPMG NMR spectra for intestinal ischemia, strangulated obstruction, and mechanical obstruction.

Predicted/Diagnosed	Intestinal Ischemia	Intestinal Strangulated Obstruction	Intestinal Mechanical Obstruction
	CPMG SPECTRA (Cross Validation)		
Predicted as Intestinal ischemia	6	1	0
Predicted as Intestinal Strangulated Obstruction	0	5	0
Predicted as Intestinal Mechanical Obstruction	0	0	11
Accuracy (%)	95.7	90.7	100

## Supplementary Materials

Supplementary materials can be found at http://www.mdpi.com/1422-0067/19/11/3288/s1.

## Abbreviations

LC-MS	Liquid Chromatography–Mass Spectrometry
NMR	Nuclear Magnetic Resonance
NOESY	Nuclear Overhauser Effect Spectroscopy
CPMG	Carr-Purcell-Meiboom-Gill
UAP	Upper Abdominal Pain
DAIP	Diffuse Abdominal/Intestinal Pain
HMDB	Human Metabolome Database
LV	Latent Variable
OPLS-DA	Orthogonal Partial Least Square–Discriminant Analysis
MVDA	Multivariate Discriminant Analysis
VIP	Variable Importance in Projection
PCA	Principal Component Analysis
PE	Phosphatidylethanolamine
PC	Phosphatidylcholine
