# Supplementary material for: Fingerprinting Acute Digestive Diseases by Untargeted NMR Based Metabolomics
Source: Int J Mol Sci. 2018 Oct 23;19(11):3288. doi: 10.3390/ijms19113288 (PMC6274841; doi:10.3390/ijms19113288)
Supplement: Supplementary file 1 [file ijms-19-03288-s001.pdf]

SUPPLEMENTARY MATERIAL

Fingerprinting acute digestive diseases by untargeted NMR based metabolomics

Panteleimon G. Takis[1], Antonio Taddei[2], Riccardo Pini[3], Stefano Grifoni[4], Francesca Tarantini[3], Paolo Bechi[2], Claudio Luchinat*[,1,5]

[1] Giotto Biotech, S.r.l, Via Madonna del Piano 6, 50019, Sesto Fiorentino, Italy
[2] Department of Surgery and Translational Medicine, School of medicine, Careggi University Hospital, Largo Brambilla 3, 50134, Florence, Italy
[3] Department of Experimental and Clinical Medicine, University of Florence, Largo Brambilla 3, 50134, Florence, Italy
[4] Department of Emergency Medicine and Surgery, Careggi University Hospital, Florence, Italy
[5] Magnetic Resonance Center (CERM), University of Florence, Via L. Sacconi 6, 50019 Sesto Fiorentino, Italy
* Correspondence should be addressed to Claudio Luchinat: claudioluchinat@cerm.unifi.it

Contents

Table of Contents
pages

NMR sample preparation and spectra acquisition 3

Figure S1. Permutation test results plot (1000 iterations) for the OPLS-DA model of the CPMG NMR serum spectra for the 2 groups of patients with upper abdominal pain (upper panel) and for the patients with diffuse abdominal/intestinal pain 4

Table S1. Probability of Model Insignificance vs. Permuted Samples for model with 2 components (500 iterations). 4

Figure S2. Loading plot of the OPLS-DA analysis from the CPMG NMR serum spectra for the 2 groups of patients with upper abdominal pain (upper panel) and for the patients with diffuse abdominal/intestinal pain 5

Figure S3. 2D Score plot of the PCA analysis for symptomatic gallstones, cholecystitis and pancreatitis serum samples based upon the ^{1}H-NMR NOESY spectra. 5

Figure S4. 3D score plot of the classification of symptomatic gallstones, cholecystitis and pancreatitis from the OPLS-DA derived model based upon serum ^{1}H-NMR NOESY spectra. 6

Figure S5. Error classification plots for OPLS-DA model LV selection of symptomatic gallstones, cholecystitis and pancreatitis diseases. 7

Table S2. Cross-Validation results (confusion matrices) and accuracies from the OPLS-DA analysis of the NOESY NMR spectra for symptomatic gallstones, pancreatitis and cholecystitis. 7

Figure S6. ^{1}H-NMR CPMG serum spectra of the 64 patients involved in this study 8

Figure S7. Different viewing angles of the 3D score plot of Fig. 5a. 9

Figure S8. The OPLS-DA analysis results along with the 2D score plots and the confusion matrices (cross-validated) of pancreatitis vs cholecystitis and symptomatic gallstones, symptomatic gallstones vs cholecystitis and pancreatitis, cholecystitis vs symptomatic gallstones and pancreatitis. 10

Figure S9. Loadings plot of the OPLS-DA model of symptomatic gallstones, cholecystitis and pancreatitis diseases derived from NOESY and CPMG spectra. 11

Figure S10. Loading plots of the OPLS-DA analysis from NOESY spectra focused on the variables-NMR buckets (metabolites) that contribute to the diseases discrimination. 12

Figure S11. Loading plots of the OPLS-DA analysis from CPMG spectra focused on the variables-NMR buckets (metabolites) that contribute to the diseases discrimination. 13

Figure S12. Boxplots and calculated probability values (p) from Kruskal–Wallis non-parametric analysis of variance test of the weighted variables (metabolites' NMR signals) for the diseases classification. 14

Figure S13. Boxplots and calculated probability values (p) from Kruskal–Wallis non-parametric analysis of variance test of the weighted variables (metabolites' NMR signals) for the diseases classification. 15

Figure S14. 2D Score plot of the OPLS-DA classification of symptomatic gallstones, cholecystitis and pancreatitis diseases based upon the ^{1}H-NMR diffusion patients' serum spectra. 16

Figure S15. Error classification plots for OPLS-DA model LV selection of symptomatic gallstones, cholecystitis and pancreatitis diseases from diffusion spectra. **16**

Table S3. The cross-validated values of sensitivity, specificity and accuracy of symptomatic gallstones, cholecystitis and pancreatitis diseases groups in the OPLS-DA derived model based upon serum ^{1}H-NMR diffusion spectra. **17**

Table S4. Prediction probabilities of test data by the use of the NOESY and CPMG models, for the symptomatic gallstones, cholecystitis and pancreatitis diseases. **17**

Figure S16. CPMG and NOESY prediction plots for the test data used. **18**

Figure S17. 3D score plot of the classification of symptomatic gallstones, cholecystitis and pancreatitis from the OPLS-DA derived model based upon serum ^{1}H-NMR NOESY and diffusion spectra. **19**

Figure S18. Error classification plots for OPLS-DA model LV selection of intestinal ischemia, intestinal strangulated obstruction and intestinal mechanical obstruction diseases. **20**

Table S5. Cross-Validation results (confusion matrices) and accuracies from the OPLS-DA analysis of NOESY and diffusion NMR spectra for intestinal ischemia, strangulated obstruction and mechanical obstruction. **21**

Figure S19. The OPLS-DA analysis results along with the 2D score plots and the confusion matrices (cross-validated) of intestinal ischemia vs intestinal mechanical and strangulated obstruction, intestinal strangulated obstruction vs intestinal ischemia and mechanical obstruction, intestinal mechanical obstruction vs intestinal ischemia and strangulated obstruction. **22**

Figure S20. Latent Variable (LV) first components of the OPLS-DA models of intestinal ischemia, intestinal strangulated obstruction and intestinal mechanical obstruction diseases. **23**

Figure S21. Loading plots of the OPLS-DA analysis from NOESY spectra focused on the variables-NMR buckets (metabolites) that contribute to the diseases discrimination. **24**

Figures S22-S23. Loading plots of the OPLS-DA analysis from CPMG spectra focused on the variables-NMR buckets (metabolites) that contribute to the diseases discrimination. **25–26**

Table S6. Prediction probabilities of test data by the use of the CPMG model, for the intestinal ischemia, intestinal strangulated and mechanical obstruction diseases. **27**

Figure S24. The CPMG, NOESY and diffusion models prediction plots for the test data used. **28**

NMR sample preparation and spectra acquisition

Frozen serum samples were thawed at room temperature and 300 µl of each sample were added to a total of 300 µl of a sodium phosphate buffer at pH 7.4 (75 mM Na_2HPO_4, 20 % (v/v) D2O, 0.025 % (v/v) NaN_3 and 0.8 % (w/v) sodium trimethylsilyl[2,2,3,3-2^2H_4]propionate (TMSP)). From the mixture, 450 µl were transferred into a 4.25 mm NMR disposable tube, acquired from Bruker BioSpin srl.

^{1}H-NMR experiments were conducted using a Bruker 600 MHz metabolic profiler (Bruker BioSpin) operating at 600.13 MHz proton Larmor frequency and equipped with a 5 mm CPTCI ^{1}H-^{13}C-^{31}P and ^{2}H-decoupling cryoprobe including a z-axis gradient coil, an automatic tuning-matching and an automatic sample changer. A BTO 2000 thermocouple served for temperature stabilization at the level of approximately 0.1 K at the sample. Before measurement, samples were kept for at least 3 min inside the NMR probehead for temperature equilibration (310 K). Three mono-dimensional (1D) spectra were acquired: a) A standard Nuclear Overhauser Effect Spectroscopy (NOESY) (1) 1D presat pulse sequence (noesygppr1d.comp, Bruker BioSpin) with 98304 data points, 18028 Hz spectral width, acquisition time of 2.7 s, relaxation delay of 4 s and mixing time of 0.1 s. NOESY spectra contain ^{1}H-NMR signals from small molecular weight metabolites as well as from high molecular weight molecules, such as lipids and lipoproteins. b) A standard Carr-Purcell-Meiboom-Gill (CPMG) (2) pulse sequence (cpmgpr1d.comp, Bruker BioSpin) with 73728 data points, spectral width of 12019 Hz and relaxation delay of 4 s. In this NMR experiment, ^{1}H-NMR signals of very large macromolecules are filtered out, resulting in the clearer observation of ^{1}H-NMR signals from small molecular weight metabolites than in NOESY spectra. c) A standard DIFFUSION-EDITED (3) pulse sequence (ledbgppr2s1d.comp; Bruker BioSpin) with 98304 data points, spectral width of 18028 Hz and relaxation delay of 4 s. In this experiment, mostly ^{1}H-NMR signals from large molecules are observed. For all NMR spectra 64 scans were acquired. Each one of the experiments is performed within < 5 min.

NMR acquisition free induction decays (FIDs) were multiplied by an exponential function equivalent to 1.0 Hz line-broadening factor before applying Fourier transform, and all transformed spectra were automatically corrected for phase and baseline distortions. Moreover, NOESY and CPMG spectra were calibrated by setting the glucose doublet at 5.24 ppm (this is preferred with respect to setting the TMSP singlet at 0 ppm due to TMSP interaction with serum albumin). By the use of AMIX software (Bruker Biospin), each spectrum was divided into 0.02 ppm chemical shift bins ranging from 0.3 to 9.0 ppm, and the 4.4 to 5.0 ppm spectral region around the suppressed water ^{1}H-NMR signal was excluded. Total area normalization was applied upon the remaining spectral buckets.

References

1. Mckay, RT. How the 1D-NOESY suppresses solvent signal in metabonomics NMR spectroscopy: An examination of the pulse sequence components and evolution. Concepts Magn Reson Part A Bridg Educ Res 2011;38A:197-220.
2. Carr, HY, Purcell, EM. Effects of Diffusion on Free Precession in Nuclear Magnetic Resonance Experiments. Phys Rev 1954;94:630-8.
3. Tang H, Wang Y, Nicholson JK, et al. Use of relaxation-edited one-dimensional and two dimensional nuclear magnetic resonance spectroscopy to improve detection of small metabolites in blood plasma. Anal Biochem 2004;15:260-72.

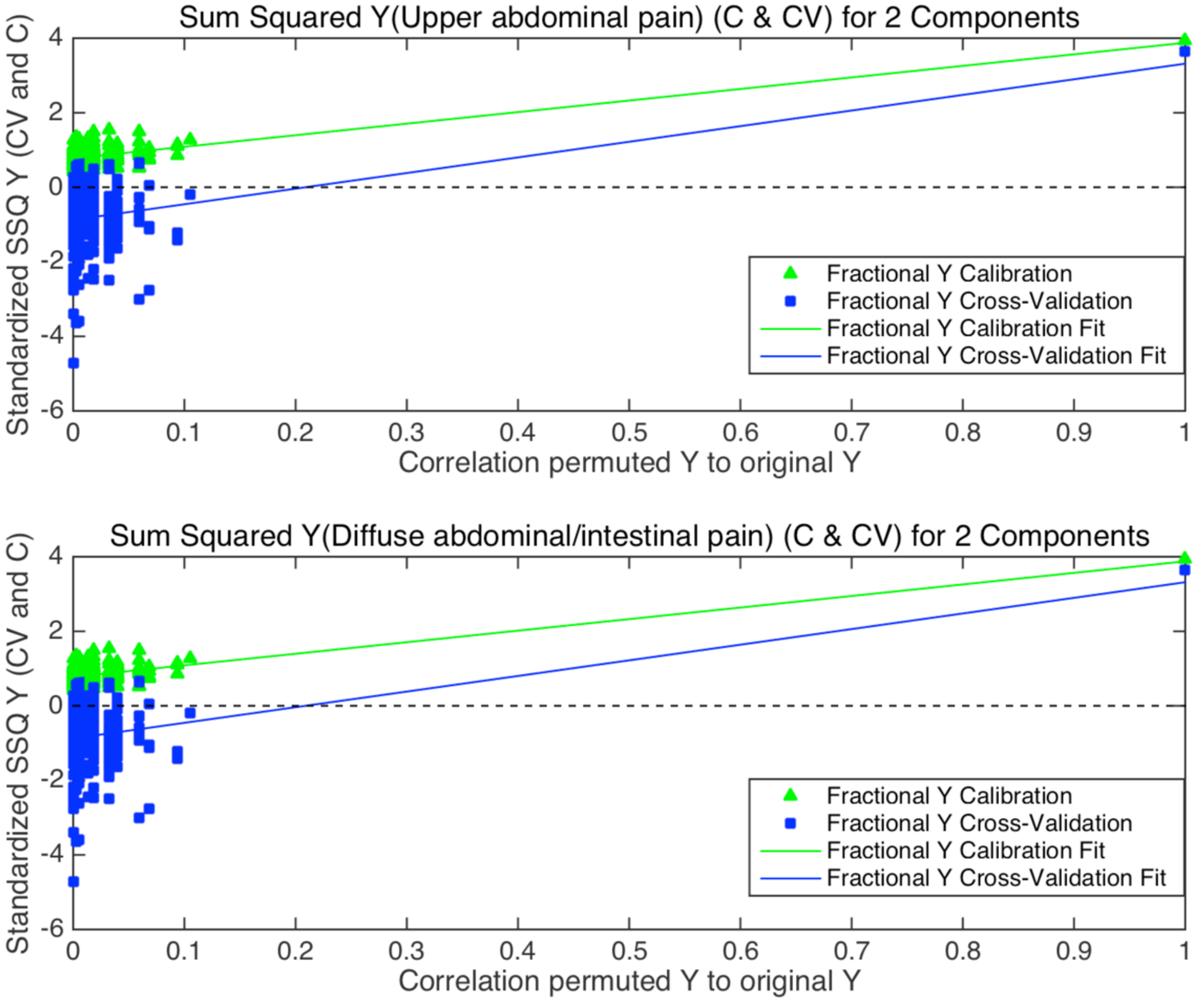

Figure S1. Permutation test results plot (500 iterations) for the OPLS-DA model of the CPMG NMR serum spectra for the 2 groups of patients with upper abdominal pain (upper panel) and for the patients with diffuse abdominal/intestinal pain (below panel).

Table S1. Probability of Model Insignificance vs. Permuted Samples for model with 2 components (500 iterations).

Classification Groups models		Wilcoxon signed test[a] (p value)	pairwise signed test[a] (p value)	randomization t-test[a] (p value)
Upper abdominal pain	Self-prediction	< 0.000	< 0.000	< 0.005
	Cross-Validated	< 0.000	< 0.000	< 0.005
Diffuse abdominal/intestinal pain	Self-prediction	< 0.000	< 0.000	< 0.005
	Cross-Validated	< 0.000	< 0.000	< 0.005

[a] p values less than 0.05 indicate that the model is significant at the 95% confidence level.

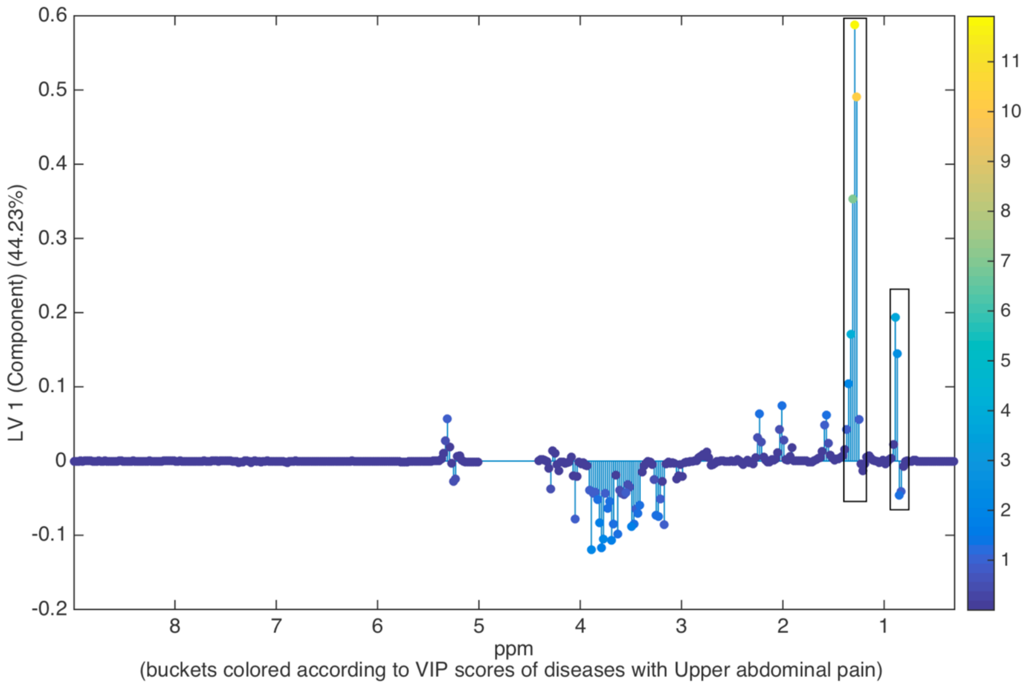

Figure S2. Loading plot of the OPLS-DA analysis from the CPMG NMR serum spectra for the 2 groups of patients with upper abdominal pain (upper panel) and for the patients with diffuse abdominal/intestinal pain, focused on the variables NMR buckets (metabolites signals) that contribute to the 2 groups discrimination. The brighter the bucket colors are, the more weighted is the bucket's (metabolite NMR signals) contribution to groups classification (weights/colors are extracted from the VIP scores of each group). The most weighted buckets are pointed in the plot by a rectangular box. These signals corresponded to the fatty acids signals in a serum NMR profile.

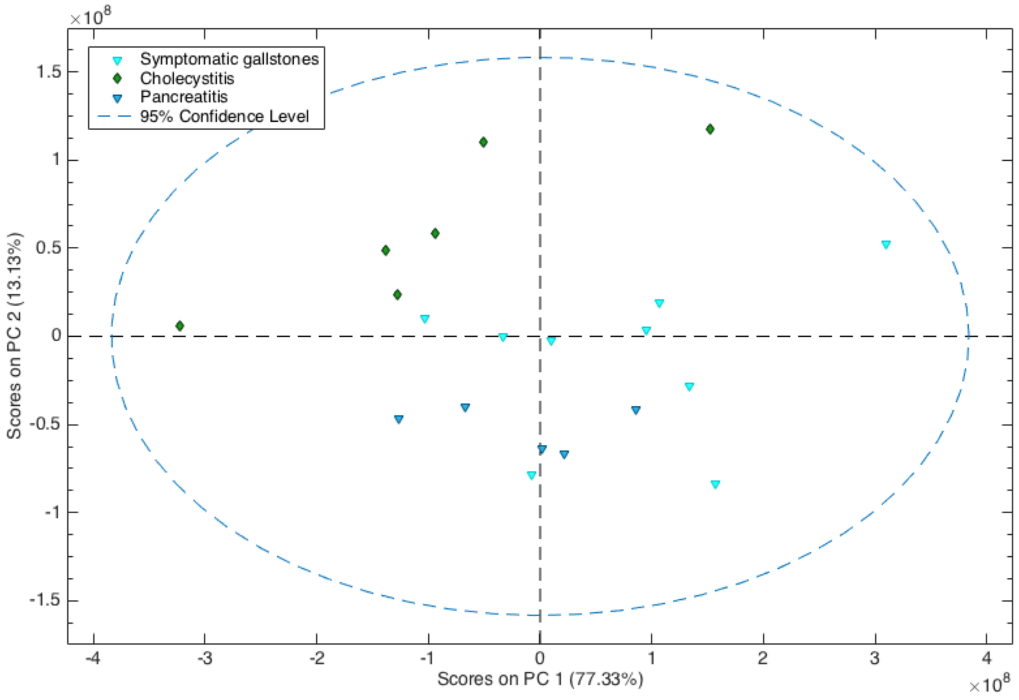

Figure S3. 2D Score plot of the PCA analysis for symptomatic gallstones, cholecystitis and pancreatitis serum samples based upon the ^{1}H-NMR NOESY spectra.

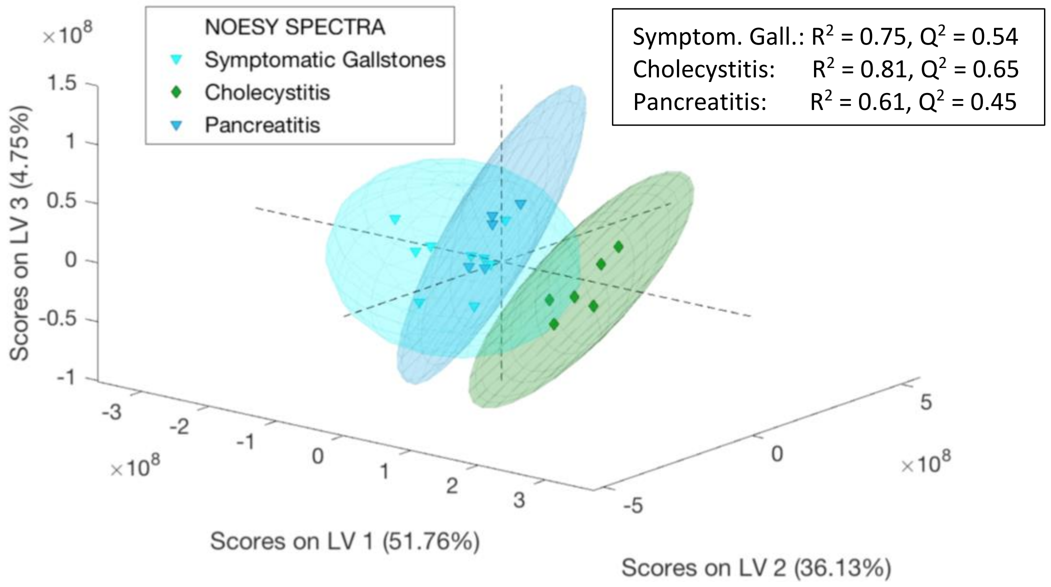

Figure S4. 3D score plot of the classification of symptomatic gallstones, cholecystitis and pancreatitis from the OPLS-DA derived model based upon serum ^{1}H-NMR NOESY spectra, along with the R^2 and Q^2 values. The score plot has been constructed after the OPLS-DA analysis of the serum spectra and the 3 first latent variables (LVs) components were used (the 3rd one is the orthogonal component), which were also used for the final model production, since after 3rd component the classification error in the model was increasing (see below Figure S5a). Except for the three groups samples distribution, the 95 % confidence of each group is depicted.

a

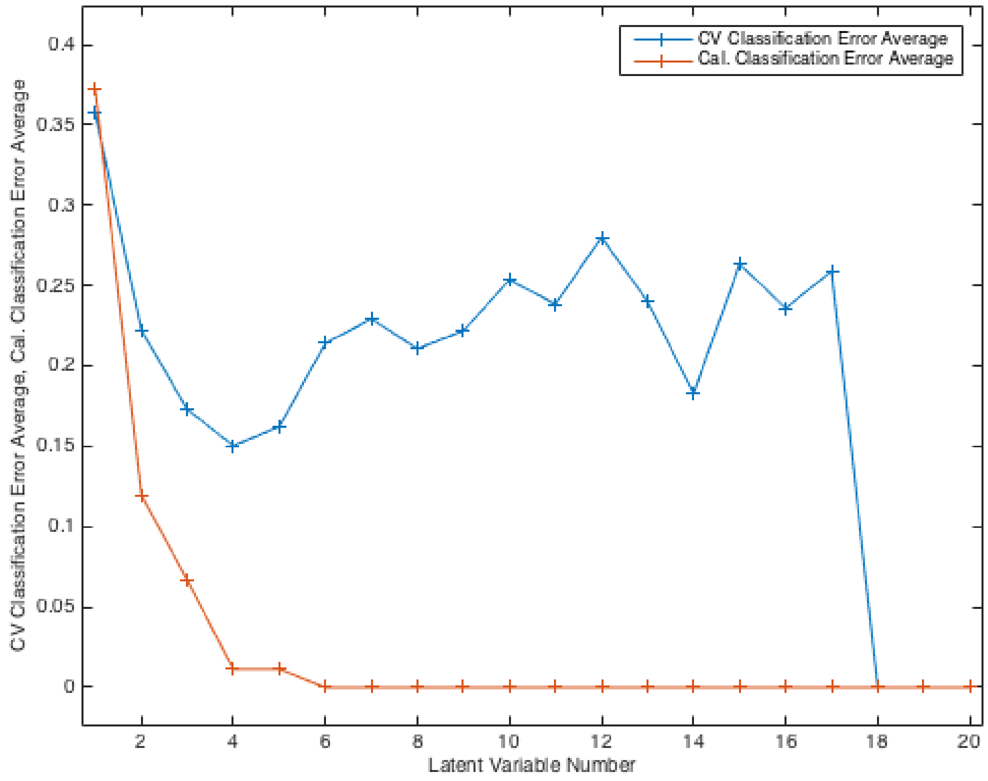

b

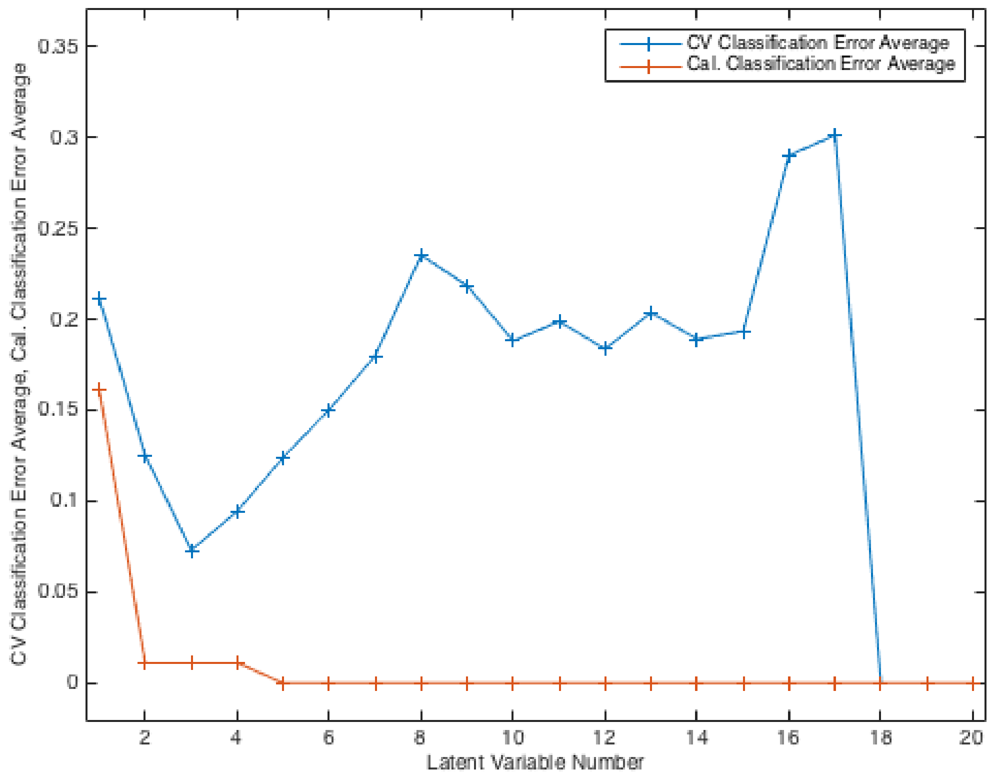

Figure S5. Error classification plots for OPLS-DA model LV selection of symptomatic gallstones, cholecystitis and pancreatitis diseases. **a)** NOESY, **b)** CPMG spectra. Blue line corresponds to the 1-[mean(Accuracy)] via Cross validation (CV) and orange line to the 1-[mean(Accuracy)] without Cross validation (Cal.).

Table S2. Cross-Validation results (confusion matrices) and accuracies from the OPLS-DA analysis of the NOESY NMR spectra for symptomatic gallstones, pancreatitis and cholecystitis.

	Symptomatic Gallstones	Cholecystitis	Pancreatitis
	NOESY SPECTRA (Cross validation)		
Predicted as Symptomatic Gallstones	8	0	1
Predicted as Cholecystitis	1	6	0
Predicted as Pancreatitis	0	0	4
Accuracy (%)	89.8	91.7	86.6

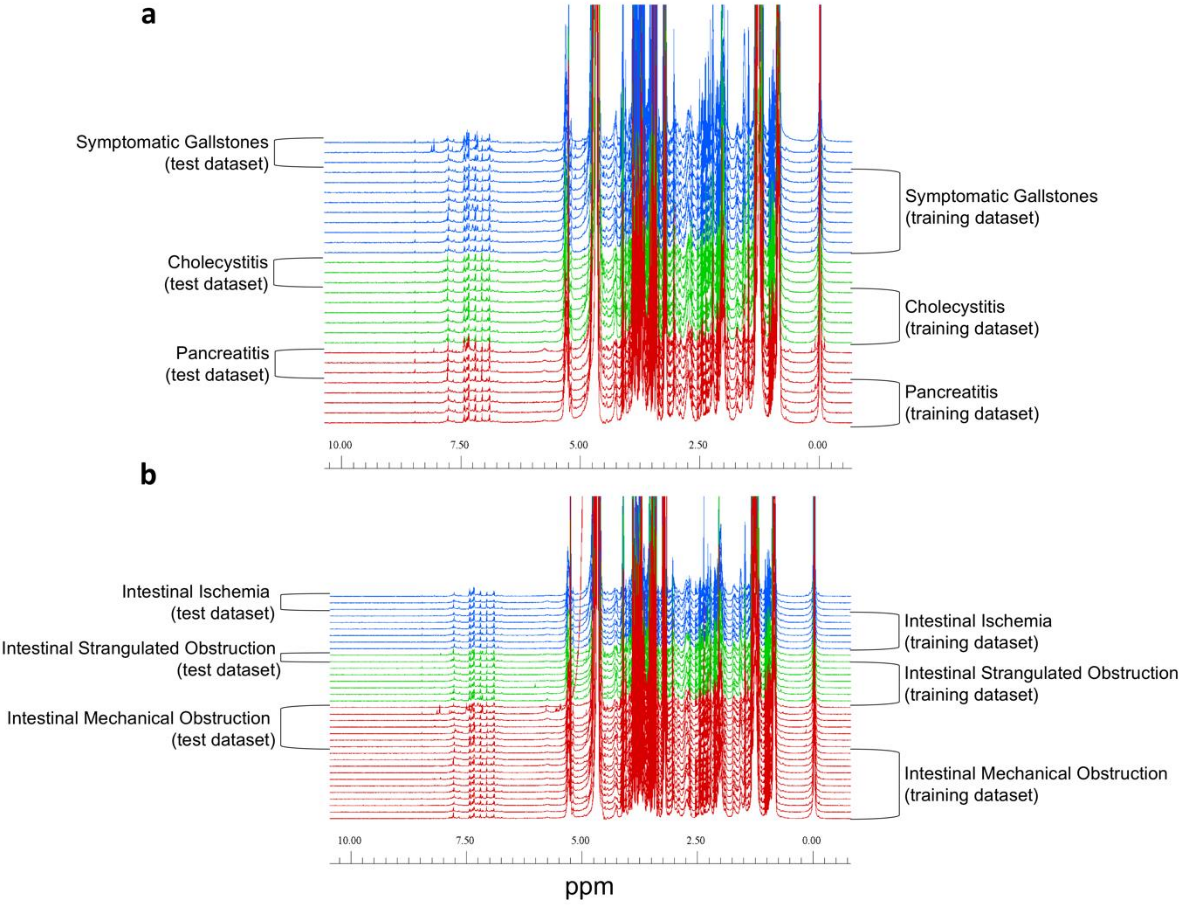

Figure S6. ^{1}H-NMR CPMG serum spectra of the 64 patients involved in this study: **a)** 29 serum spectra of patients exhibiting symptomatic gallstones, cholecystitis and pancreatitis and **b)** 35 serum spectra of patients exhibiting intestinal ischemia, intestinal strangulated obstruction and intestinal mechanical obstruction.

a

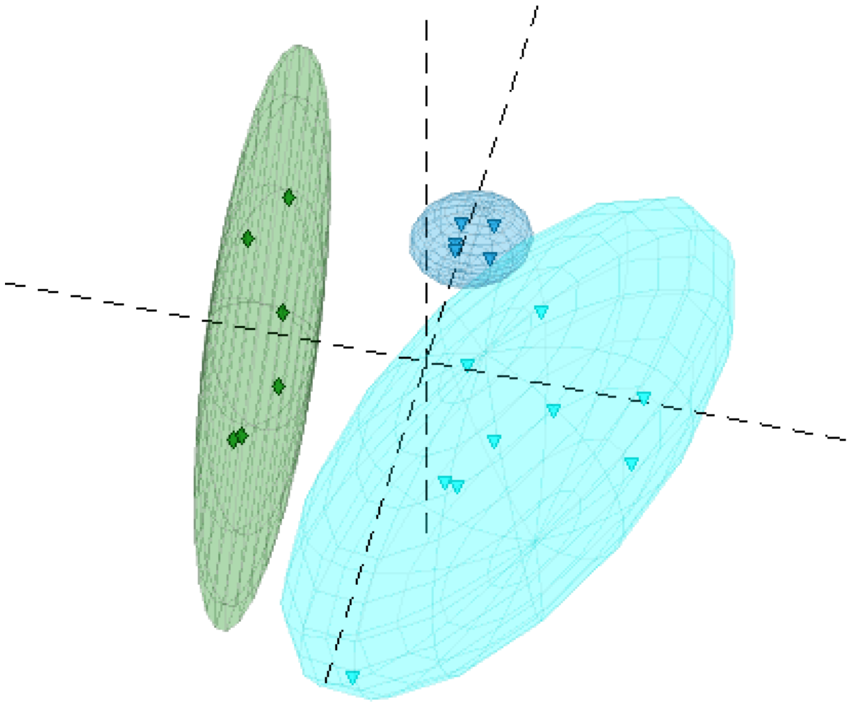

b

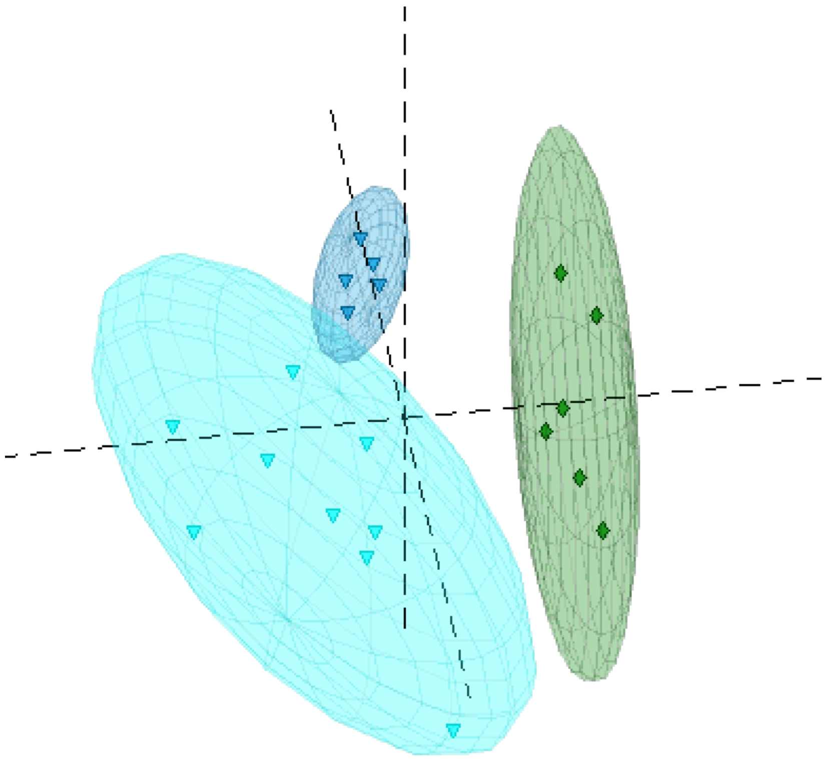

Figure S7. Different viewing angles of the 3D score plot of Figure 5a.

a

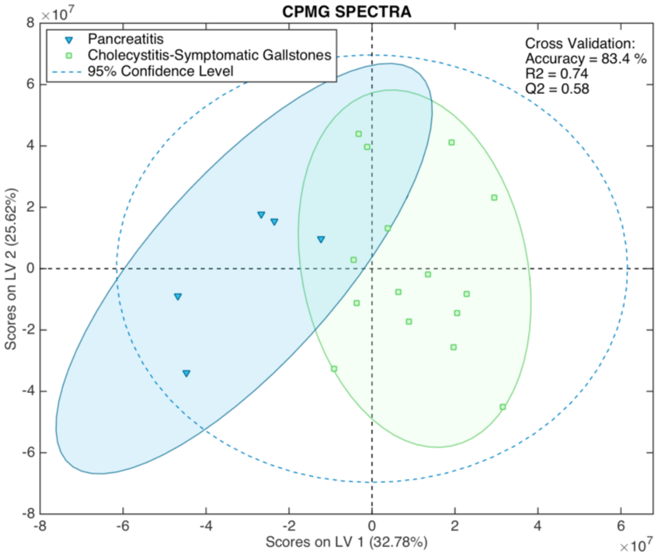

CPMG SPECTRA (Cross Validation)	Pancreatitis	Symptomatic Gallstones + Cholecystitis
Predicted as Pancreatitis	4	3
Predicted as Symptomatic Gallstones + Cholecystitis	1	12

b

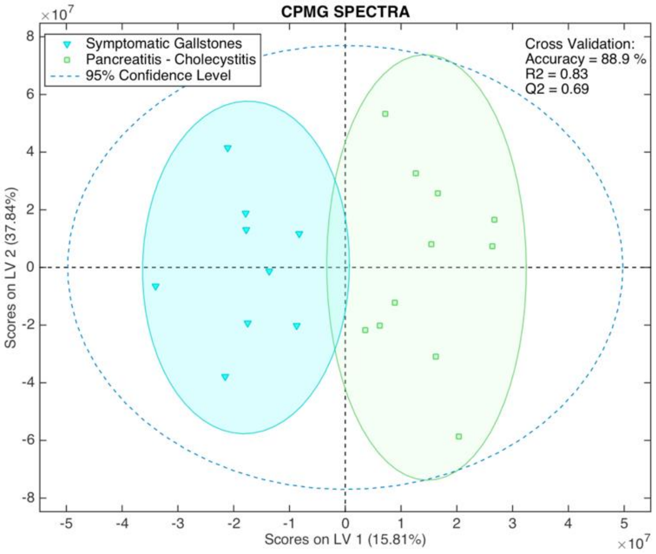

CPMG SPECTRA (Cross Validation)	Symptomatic Gallstones	Pancreatitis + Cholecystitis
Predicted as Symptomatic Gallstones	8	0
Predicted as Pancreatitis + Cholecystitis	1	11

c

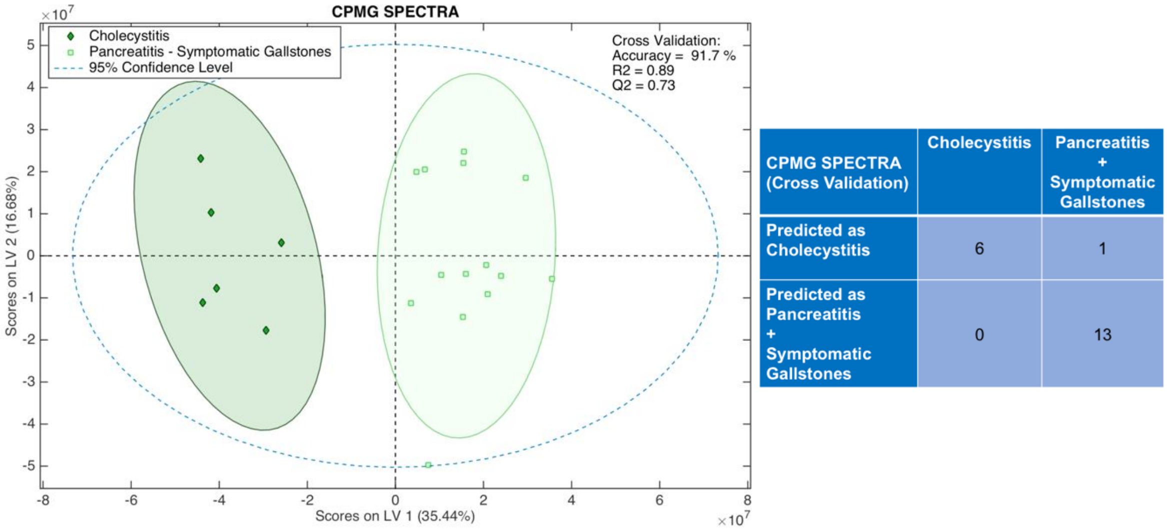

CPMG SPECTRA (Cross Validation)	Cholecystitis	Pancreatitis + Symptomatic Gallstones
Predicted as Cholecystitis	6	1
Predicted as Pancreatitis + Symptomatic Gallstones	0	13

Figure S8. The OPLS-DA analysis results along with the 2D score plots (2nd component is orthogonal) and the confusion matrices (cross-validated) of **a)** pancreatitis vs cholecystitis and symptomatic gallstones, **b)** symptomatic gallstones vs cholecystitis and pancreatitis, **c)** cholecystitis vs symptomatic gallstones and pancreatitis.

.

a

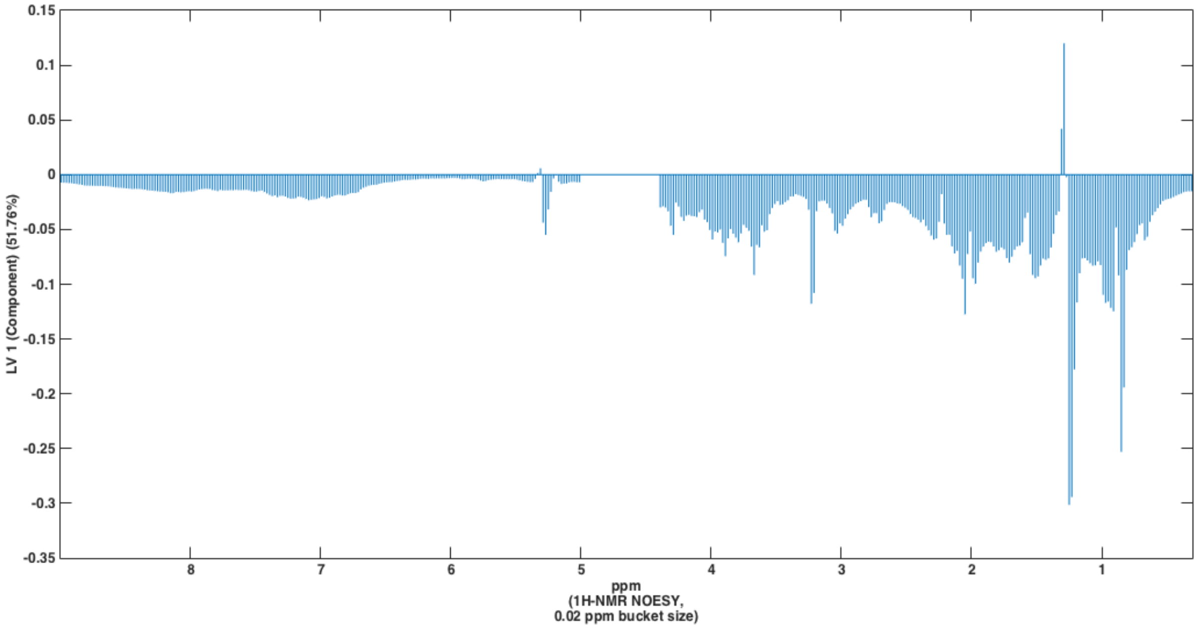

b

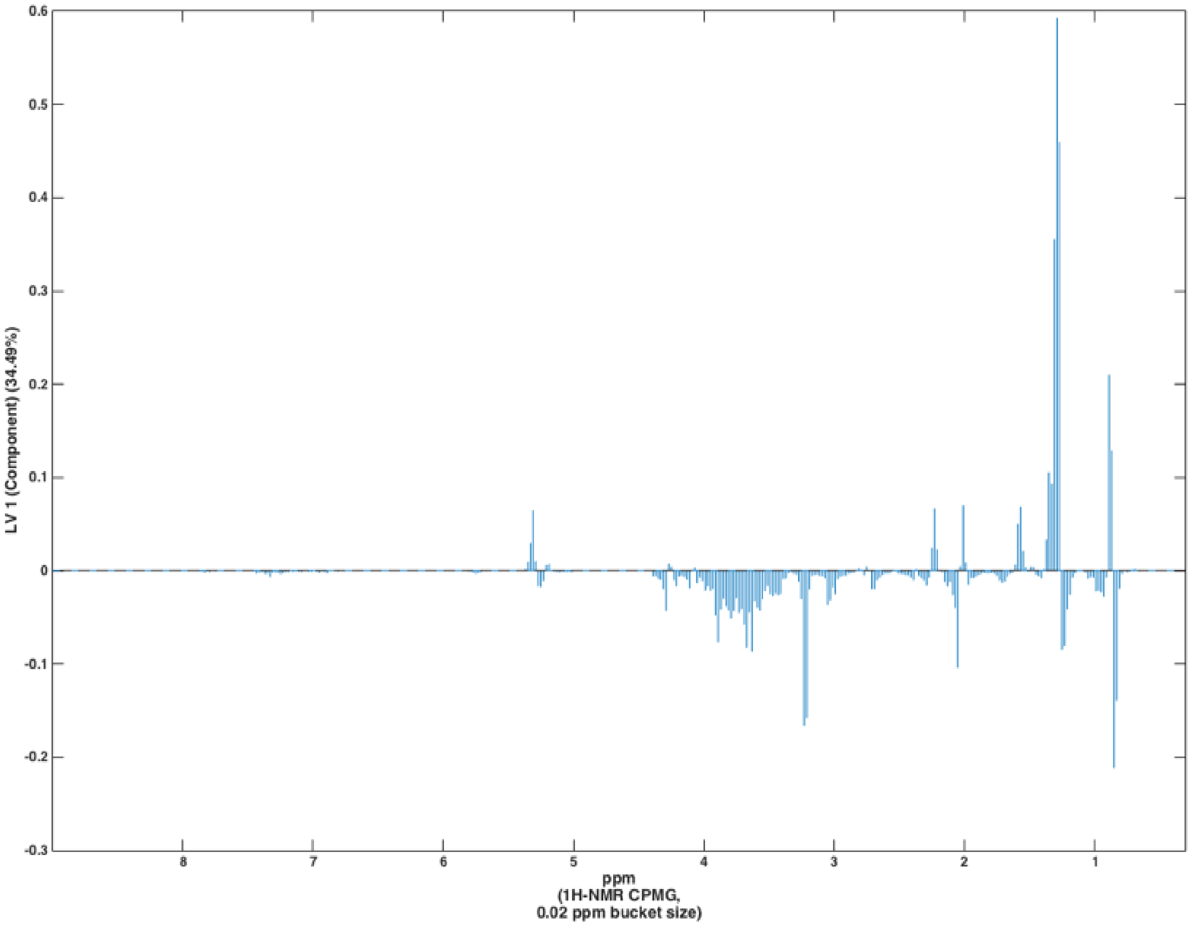

Figure S9. Loadings plot of the OPLS-DA model of symptomatic gallstones, cholecystitis and pancreatitis diseases derived from: **a)** NOESY, **b)** CPMG spectra.

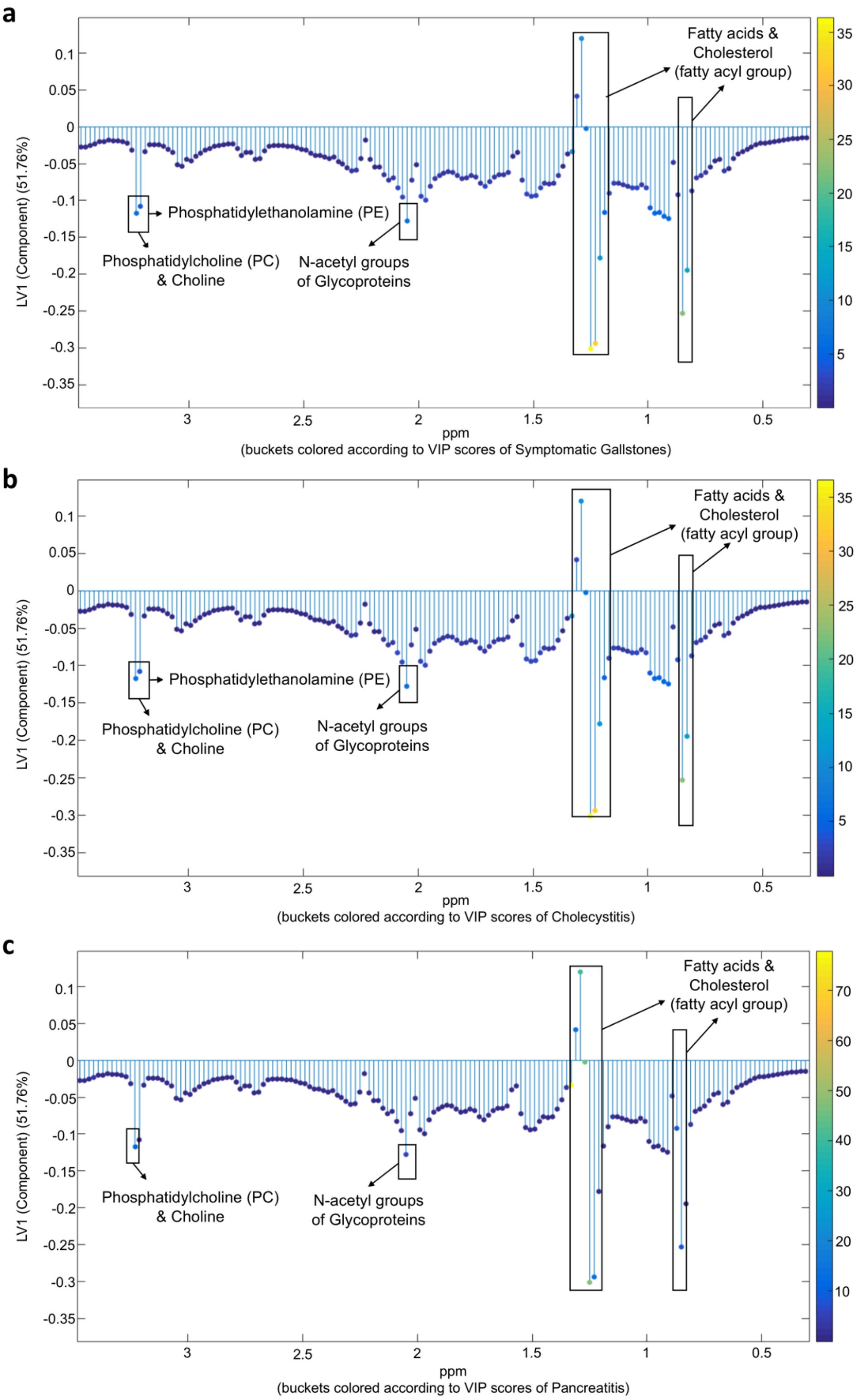

Figure S10. Loading plots of the OPLS-DA analysis from NOESY spectra focused on the variables-NMR buckets (metabolites) that contribute to the diseases discrimination. The brighter the bucket colors, the higher is the bucket's (metabolite NMR signals) contribution to each disease classification (weights/colors are extracted from the VIP scores of each disease group). a) symptomatic gallstones. b) cholecystitis. c) pancreatitis. As depicted, the metabolites contributing to the discrimination of the three diseases are fatty acids, glycoproteins and phospholipids. .

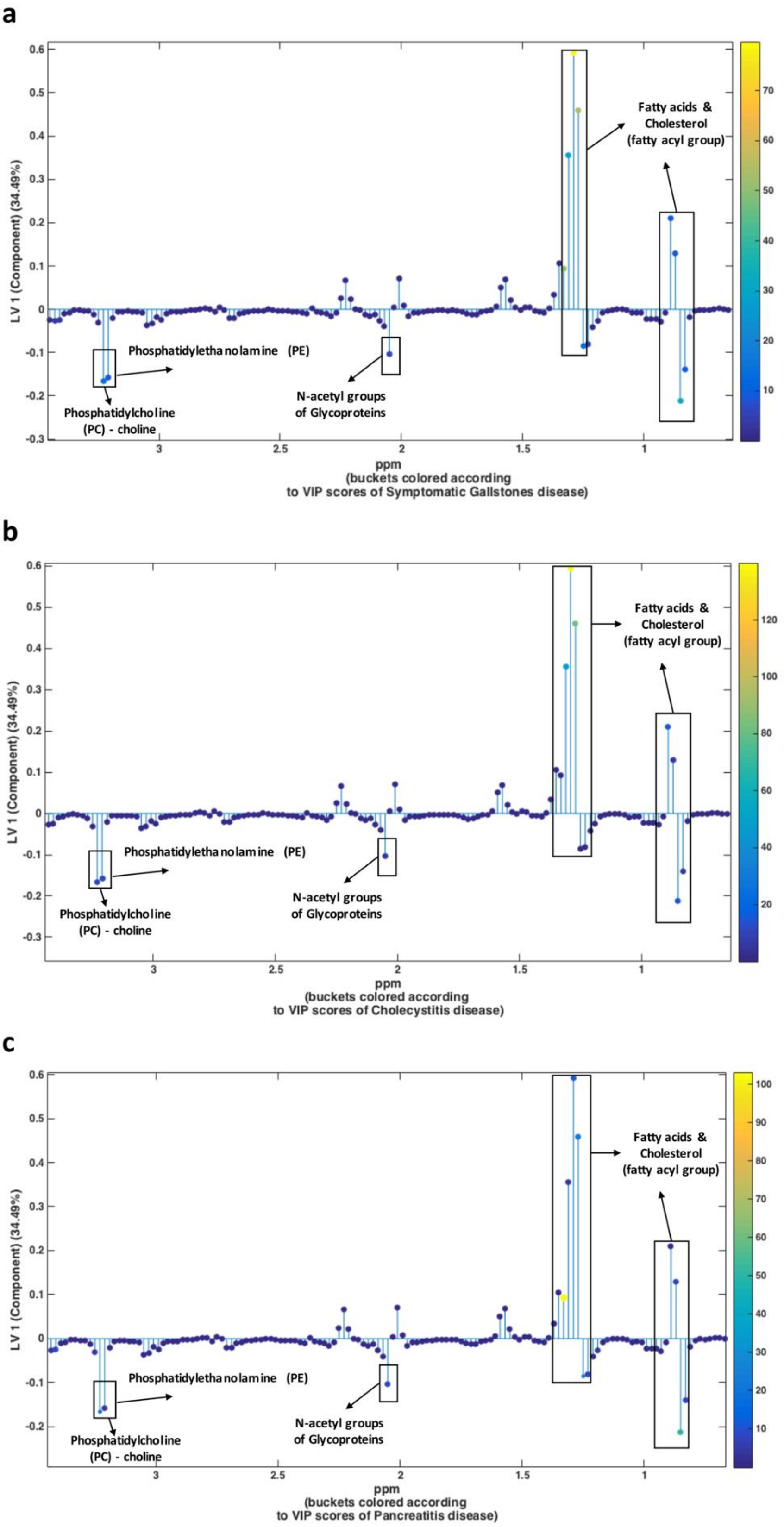

Figure S11. Loading plots of the OPLS-DA analysis from CPMG spectra focused on the variables-NMR buckets (metabolites) that contribute to the diseases discrimination. The case of: **a)** symptomatic gallstones. **b)** cholecystitis. **c)** pancreatitis. .

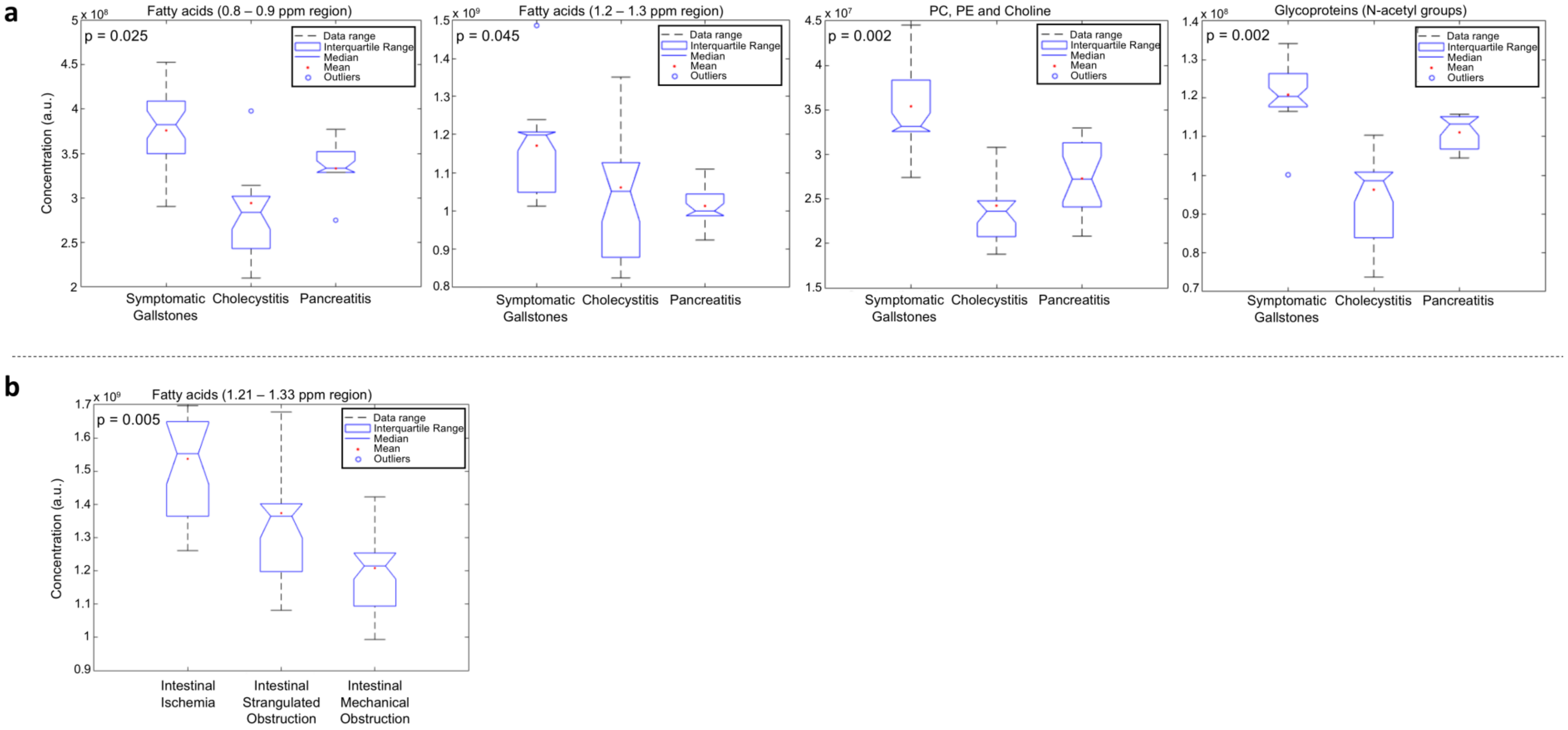

Figure S12. Boxplots and calculated probability values (p) from Kruskal–Wallis non-parametric analysis of variance test of the weighted variables (metabolites' NMR signals) for the diseases classification. Boxplots depict some of the significant metabolites for the classification ($p < 0.05$) and their concentration (in arbitrary units, a.u.) differences for each disease. The analysis was performed after integration of the NMR signals of the weighted metabolites from the NOESY spectra of the serum samples from the patients suffering from: **a)** symptomatic gallstones, cholecystitis and pancreatitis, and **b)** intestinal ischemia, intestinal strangulated obstruction and intestinal mechanical obstruction. .

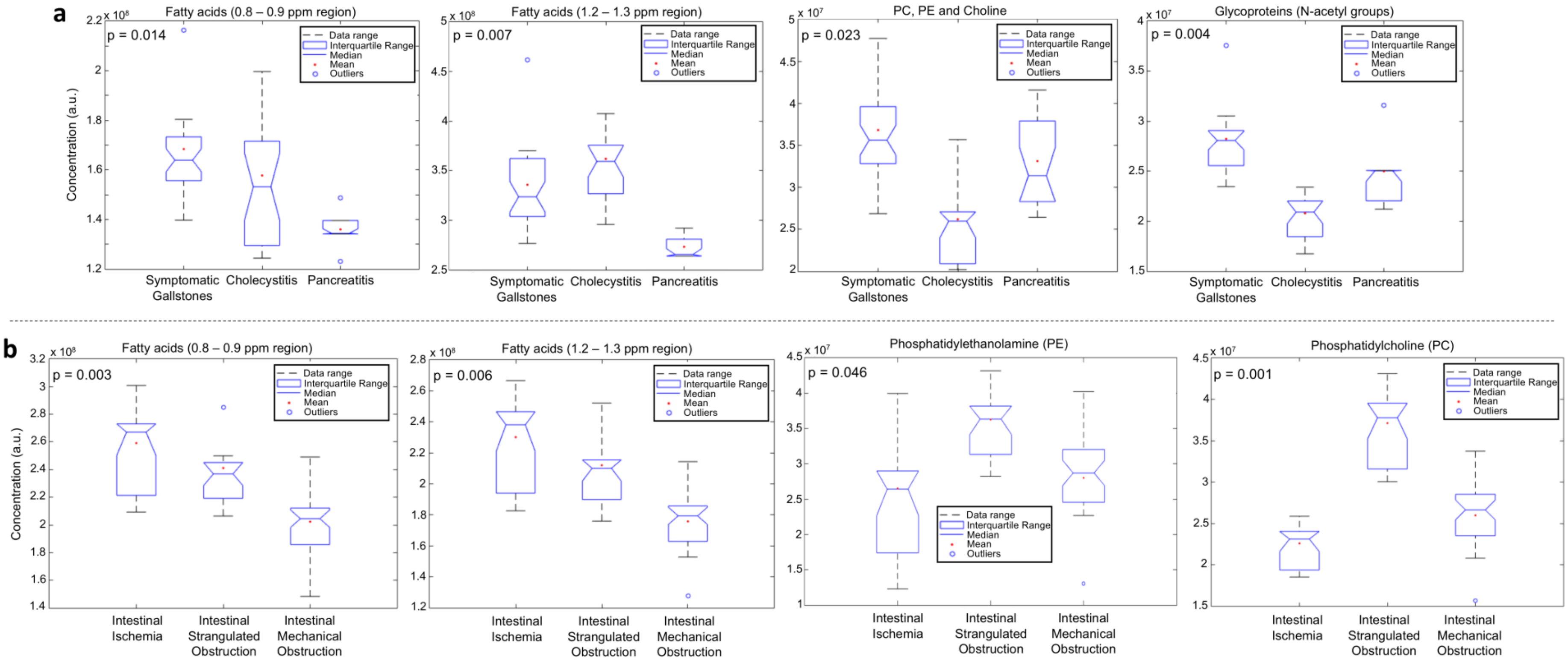

Figure S13. Boxplots and calculated probability values (p) from Kruskal–Wallis non-parametric analysis of variance test of the weighted variables (metabolites' NMR signals) for the diseases classification. Boxplots depict some of the significant metabolites for the classification ($p < 0.05$) and their concentration (in arbitrary units, a.u.) differences for each disease. The analysis was performed after integration of the NMR signals of the weighted metabolites from the CPMG spectra of the serum samples from the patients suffering from: **a)** symptomatic gallstones, cholecystitis and pancreatitis, and **b)** intestinal ischemia, intestinal strangulated obstruction and intestinal mechanical obstruction. .

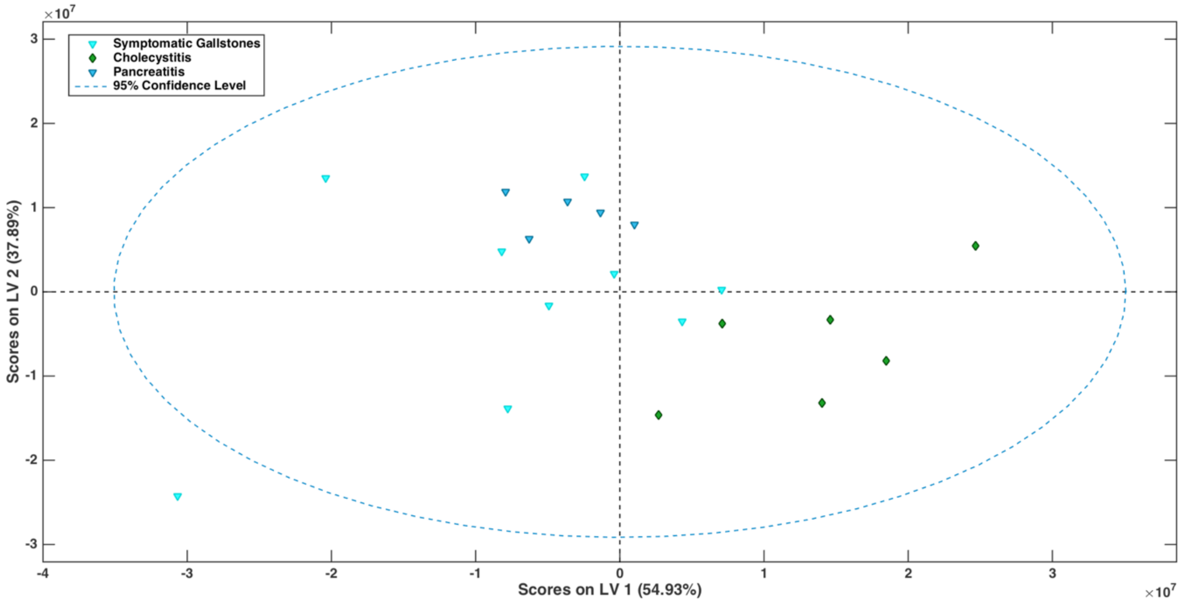

Figure S14. 2D Score plot of the OPLS-DA classification of symptomatic gallstones, cholecystitis and pancreatitis diseases based upon the ^{1}H-NMR diffusion patients' serum spectra. 2 first latent variables (LVs) components were used, which were also used for the final model production, since after 2nd component the classification error in the model were increasing (see below Figure S15).

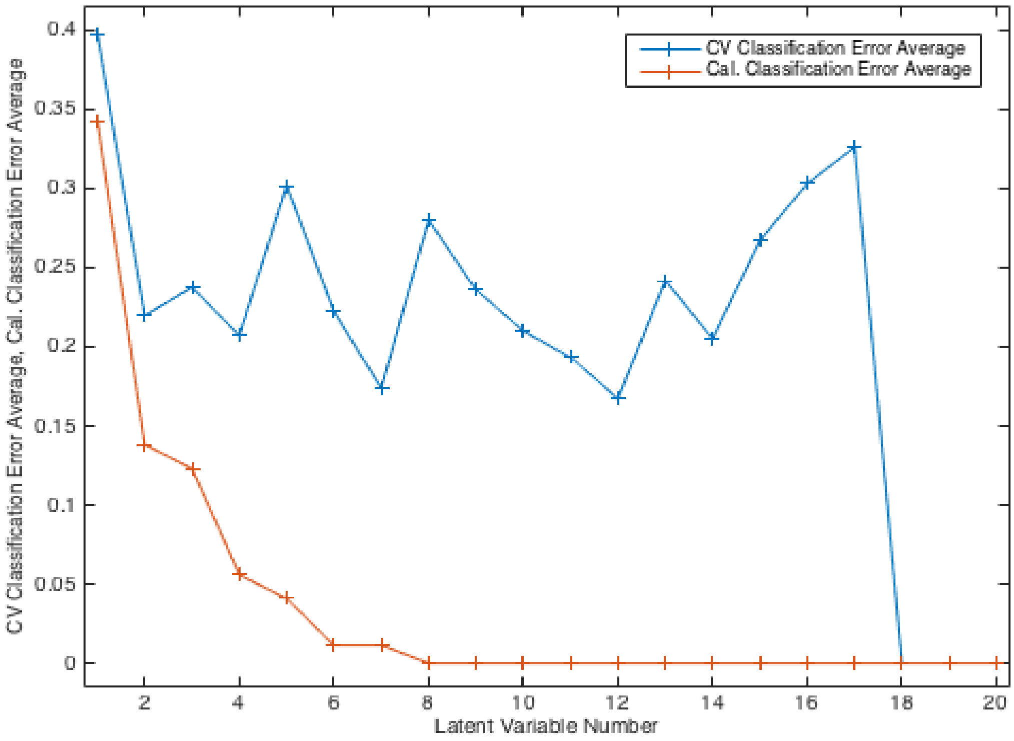

Figure S15. Error classification plots for OPLS-DA model LV selection of symptomatic gallstones, cholecystitis and pancreatitis diseases from diffusion spectra. Blue line corresponds to the 1-[mean(Accuracy)] via Cross validation (CV) and orange line to the 1-[mean(Accuracy)] without Cross validation (Cal.).

Table S3. The cross-validated values of sensitivity, specificity and accuracy of symptomatic gallstones, cholecystitis and pancreatitis diseases groups in the OPLS-DA derived model based upon serum ^{1}H-NMR diffusion spectra.

DIFFUSION SPECTRA	Symptomatic gallstones	Cholecystitis	Pancreatitis
**Sensitivity (CV)**	55.6 %	73.3 %	90.0 %
**Specificity (CV)**	63.6 %	75.7 %	80.0 %
**Accuracy (CV)**	60.0 %	74.0 %	84.0 %

Table S4. Prediction probabilities of test data by the use of the NOESY and CPMG models, for the symptomatic gallstones, cholecystitis and pancreatitis diseases. Samples with red font are erroneously predicted, and the bold highlighted values correspond to the prediction probabilities of test data for the disease that should be assigned according to medical doctors' diagnosis.

Test Samples	Medical doctors diagnosis for each data sample	Symptomatic gallstones Prediction Probability (%)	Cholecystitis Prediction Probability (%)	Pancreatitis Prediction Probability (%)
		**NOESY model**		
**1**	Pancreatitis	0.0	0.0	**98.3**
**2**	Cholecystitis	0.0	**100.0**	7.4
**3**	Pancreatitis	1.4	0.0	**98.7**
**4**	Cholecystitis	0.0	**100.0**	1.9
**5**	Symptomatic gallstones	**94.0**	97.9	0.0
**6**	Pancreatitis	1.6	0.0	**98.3**
**7**	Symptomatic gallstones	**99.8**	0.0	0.5
**8**	Symptomatic gallstones	**0.0**	98.9	0.0
**9**	Cholecystitis	0.0	**0.1**	97.1
		**CPMG model**		
**1**	Pancreatitis	0.0	0.0	**99.7**
**2**	Cholecystitis	0.0	**99.9**	0.5
**3**	Pancreatitis	19.3	0.0	**94.2**
**4**	Cholecystitis	0.0	**99.9**	0.2
**5**	Symptomatic gallstones	**100.0**	94.2	0.0
**6**	Pancreatitis	3.7	0.0	**88.7**
**7**	Symptomatic gallstones	**98.7**	0.0	46.1
**8**	Symptomatic gallstones	**100.0**	93.7	0.0
**9**	Cholecystitis	0.0	**11.5**	98.8

a

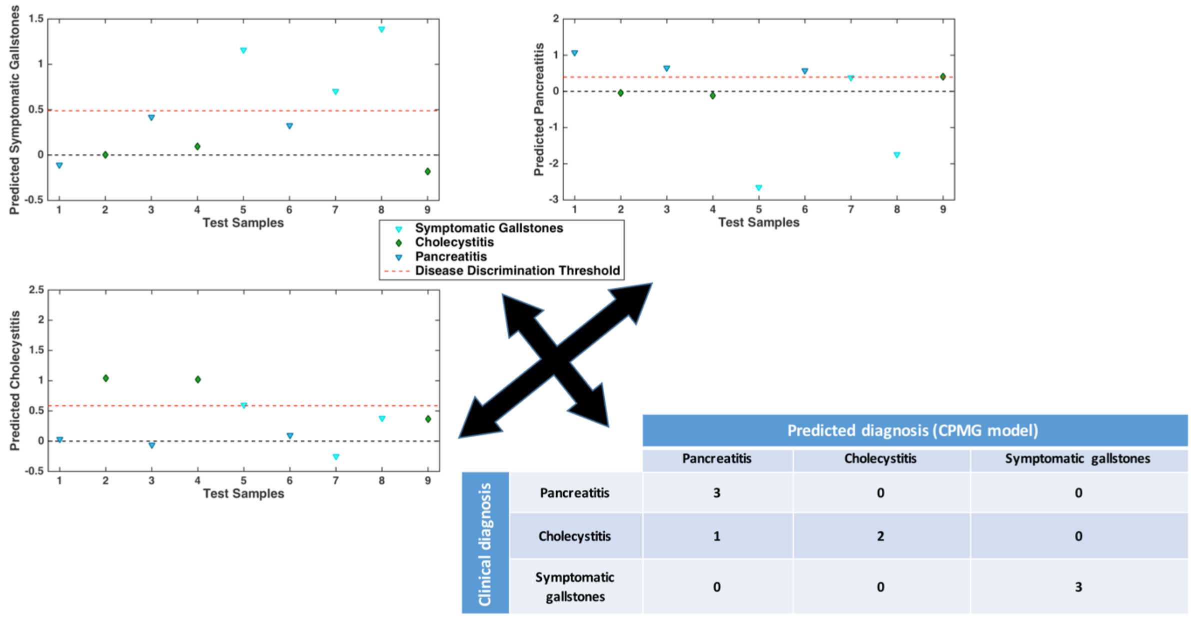

	Predicted diagnosis (CPMG model)		
Clinical diagnosis	Pancreatitis	Cholecystitis	Symptomatic gallstones
Pancreatitis	3	0	0
Cholecystitis	1	2	0
Symptomatic gallstones	0	0	3

b

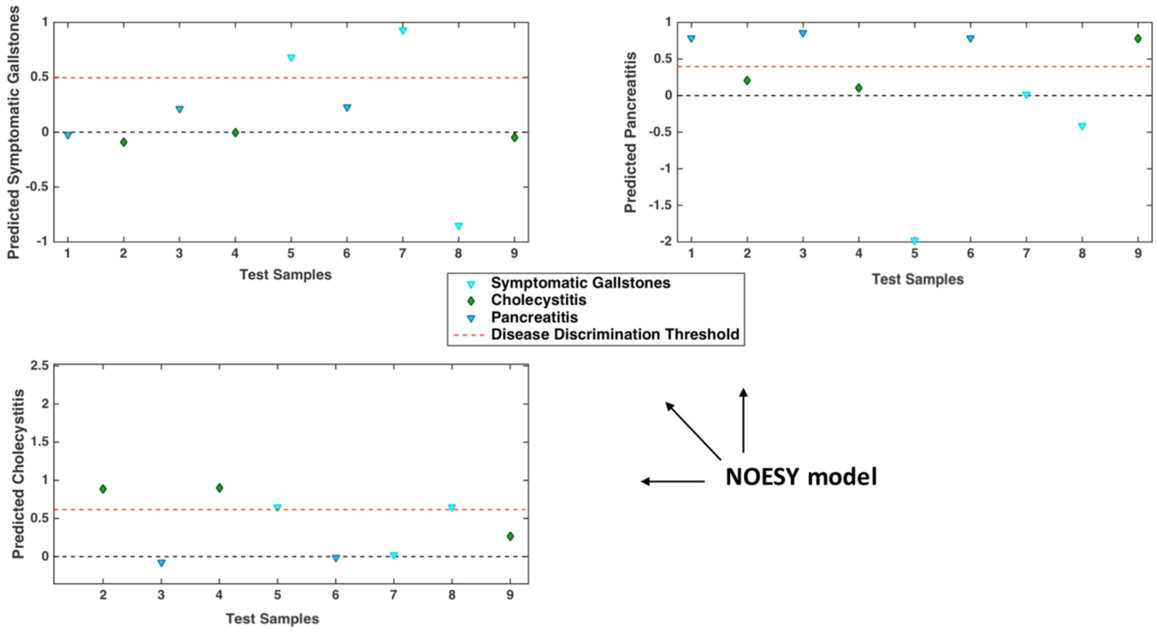

Figure S16. a) CPMG and **b)** NOESY prediction plots for the test data used. Above dashed red line (class discrimination threshold) is considered a successful prediction for each test data sample. .

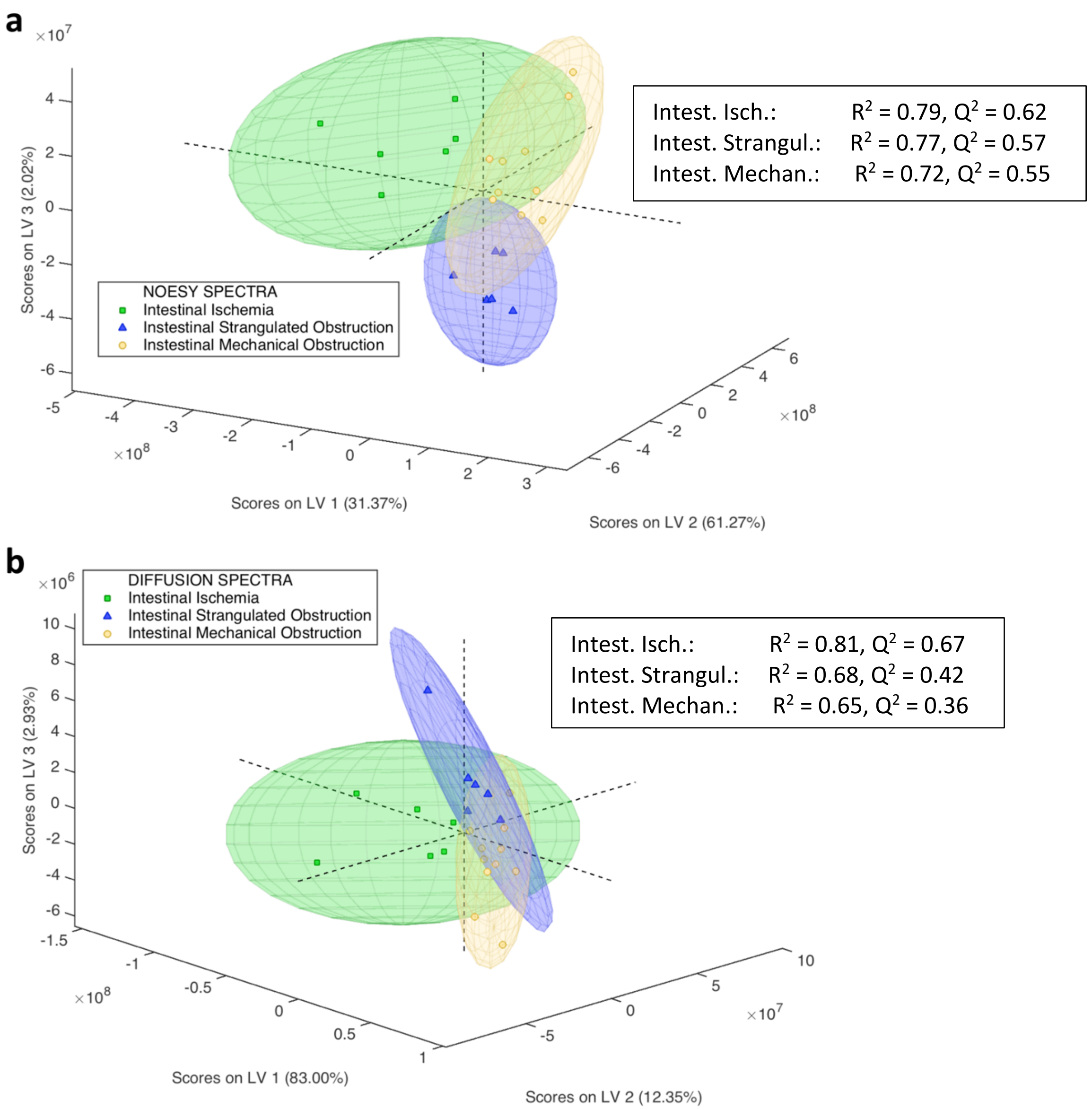

Figure S17. 3D score plot of the classification of symptomatic gallstones, cholecystitis and pancreatitis from the OPLS-DA derived model based upon serum ^{1}H-NMR **a)** NOESY and **b)** diffusion spectra, along with the R^2 and Q^2 values. The score plot has been constructed after the OPLS-DA analysis of the serum spectra and the 3 first latent variables (LVs) components were used (after the 2nd component, the rest of the components are orthogonal), (see below Figure S18a,c). Except for the three groups samples distribution, the 95 % confidence of each group is depicted. .

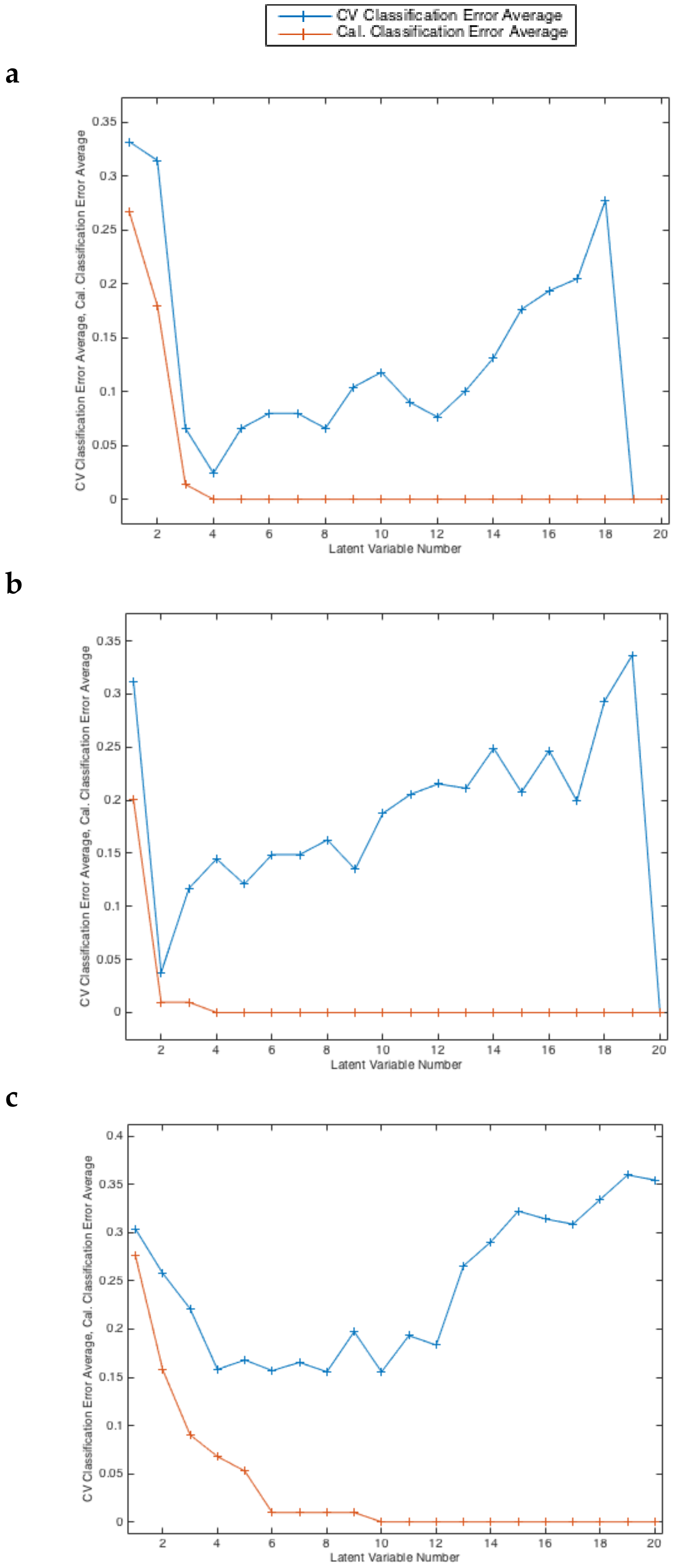

Figure S18. Error classification plots for OPLS-DA model LV selection of intestinal ischemia, intestinal strangulated obstruction and intestinal mechanical obstruction diseases. **a)** NOESY, **b)** CPMG and **c)** diffusion spectra. Blue line corresponds to the 1-[mean(Accuracy)] via Cross validation (CV) and orange line to the 1-[mean(Accuracy)] without Cross validation (Cal.).

Table S5. Cross-Validation results (confusion matrices) and accuracies from the OPLS-DA analysis of NOESY and diffusion NMR spectra for intestinal ischemia, strangulated obstruction and mechanical obstruction.

	Intestinal Ischemia	Intestinal Strangulated Obstruction	Intestinal Mechanical Obstruction
		NOESY SPECTRA (Cross validation)	
Predicted as Intestinal ischemia	5	0	0
Predicted as Intestinal Strangulated Obstruction	0	5	0
Predicted as Intestinal Mechanical Obstruction	1	1	11
Accuracy (%)	95.5	95.5	90.9
		DIFFUSION SPECTRA (Cross validation)	
Predicted as Intestinal ischemia	6	0	1
Predicted as Intestinal Strangulated Obstruction	0	4	2
Predicted as Intestinal Mechanical Obstruction	0	2	8
Accuracy (%)	95.6	82.6	78.3

a

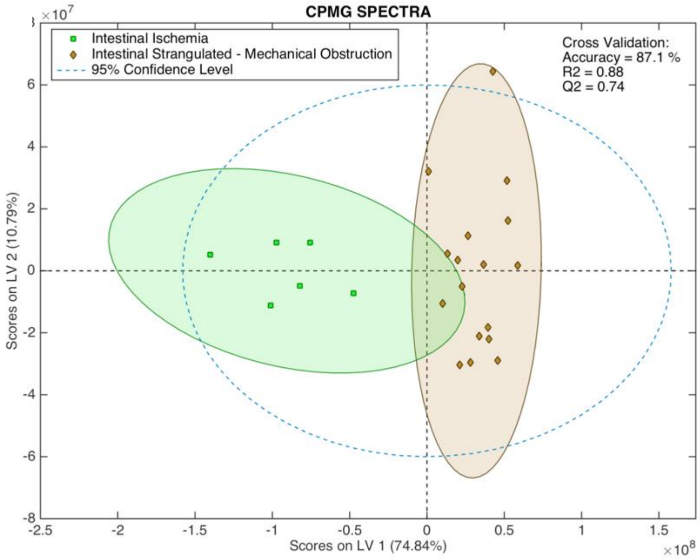

CPMG SPECTRA (Cross Validation)	Intestinal Ischemia	Intestinal Mechanical Obstruction + Intestinal Strangulated Obstruction
Predicted as Intestinal Ischemia	5	2
Predicted as Intestinal Mechanical Obstruction + Intestinal Strangulated Obstruction	1	15

b

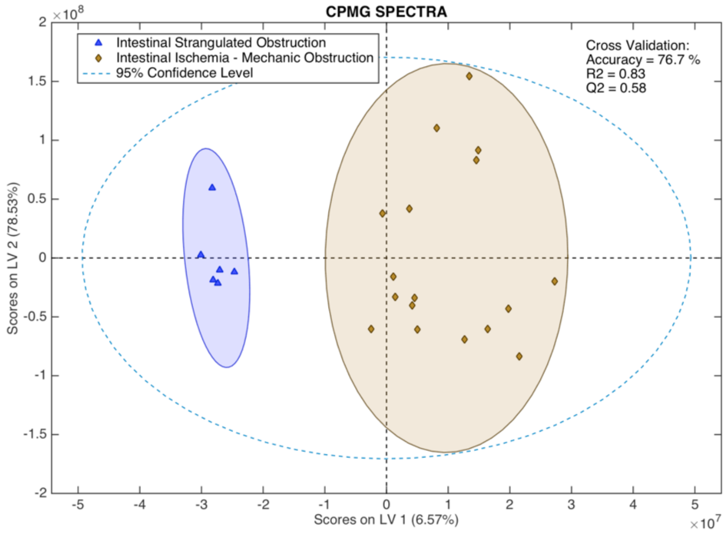

CPMG SPECTRA (Cross Validation)	Intestinal Strangulated Obstruction	Intestinal Mechanical Obstruction + Intestinal Ischemia
Predicted as Intestinal Strangulated Obstruction	4	0
Predicted as Intestinal Mechanical Obstruction + Intestinal Ischemia	2	17

c

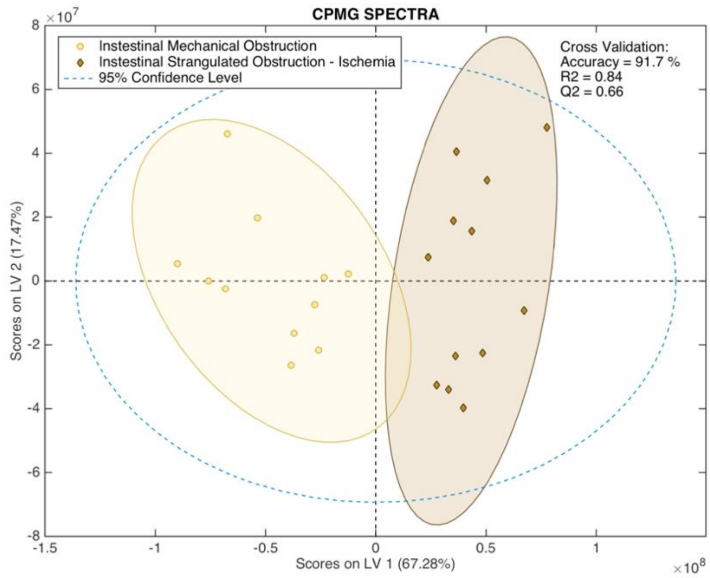

CPMG SPECTRA (Cross Validation)	Intestinal Mechanical Obstruction	Intestinal Ischemia + Intestinal Strangulated Obstruction
Intestinal Mechanical Obstruction	11	2
Predicted as Intestinal Ischemia + Intestinal Strangulated Obstruction	0	10

Figure S19. The OPLS-DA analysis results along with the 2D score plots (2nd component is orthogonal) and the confusion matrices (cross-validated) of **a)** intestinal ischemia vs intestinal mechanical and strangulated obstruction, **b)** intestinal strangulated obstruction vs intestinal ischemia and mechanical obstruction, **c)** intestinal mechanical obstruction vs intestinal ischemia and strangulated obstruction.

a

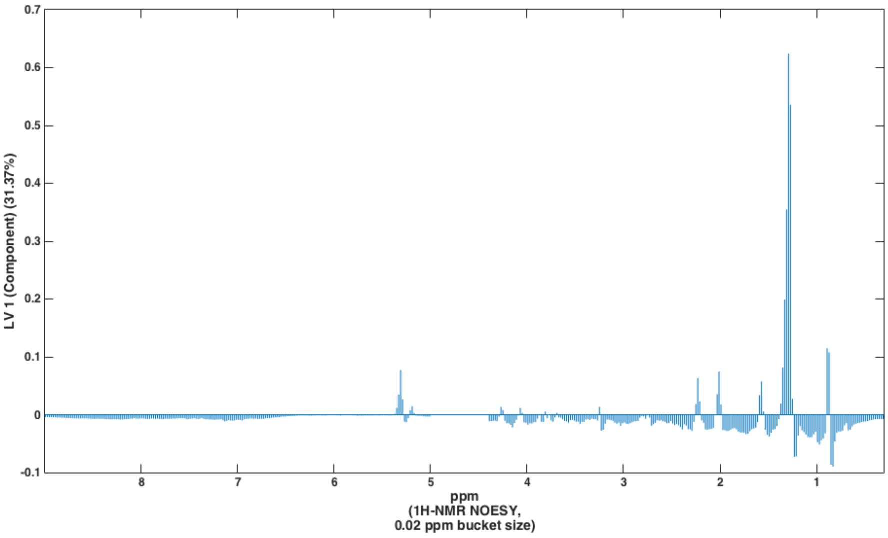

b

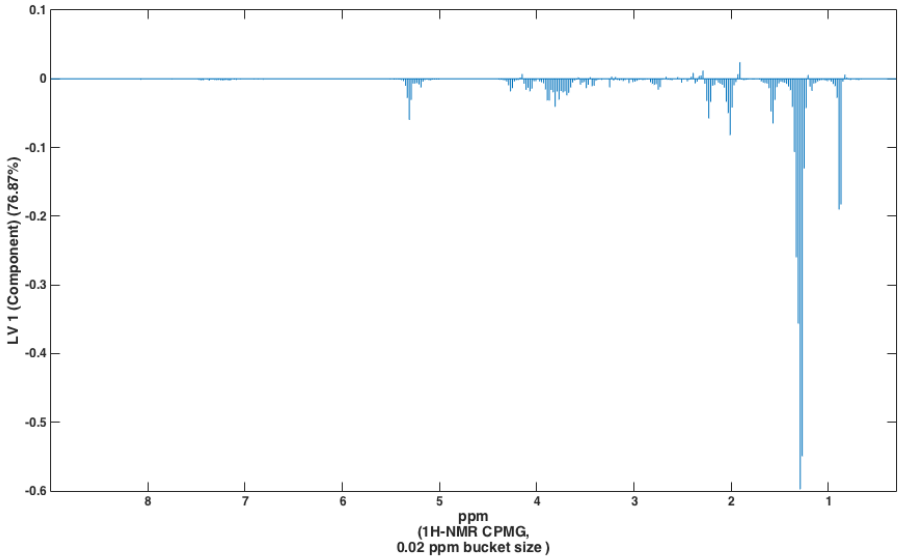

c

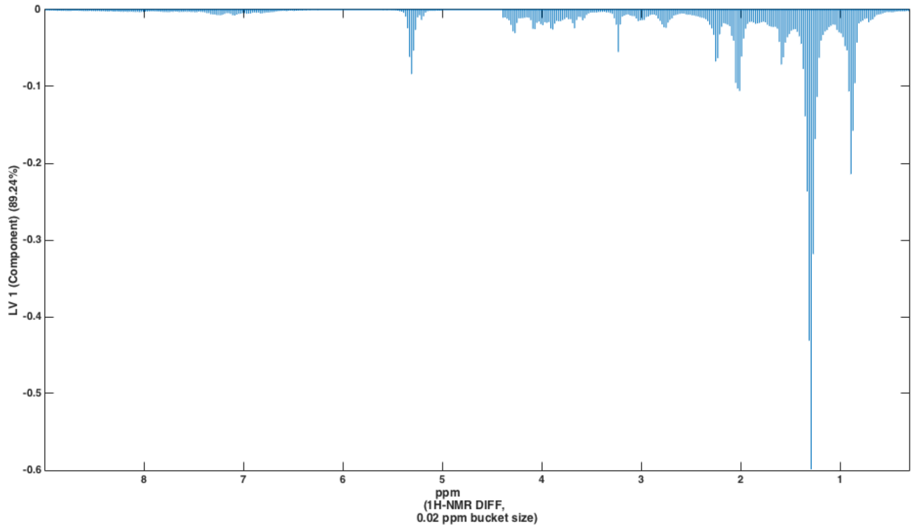

Figure S20. Latent Variable (LV) first components of the OPLS-DA models of intestinal ischemia, intestinal strangulated obstruction and intestinal mechanical obstruction diseases. **a)** NOESY, **b)** CPMG and **c)** diffusion spectra.

a

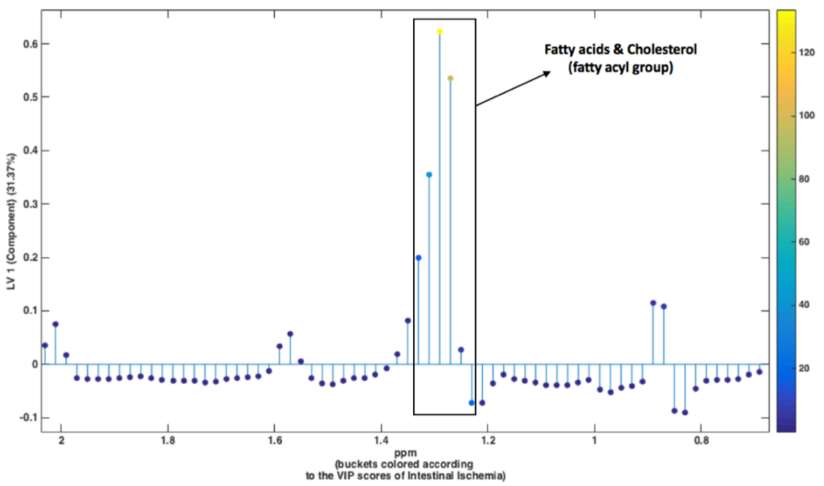

b

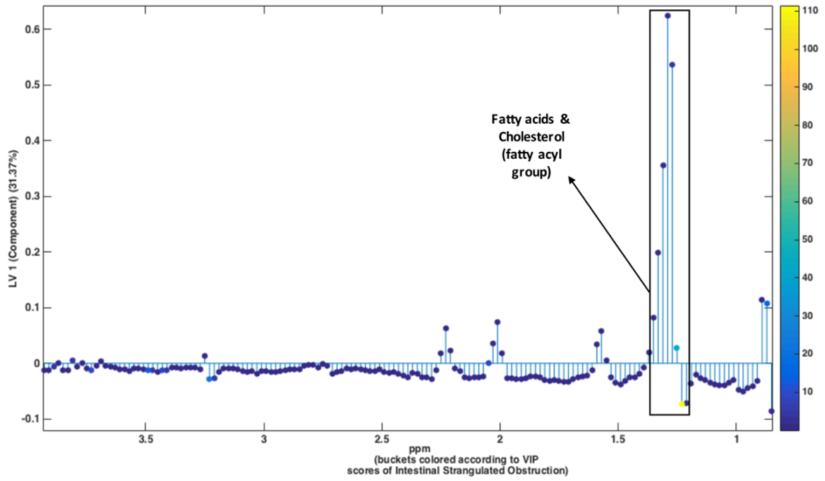

c

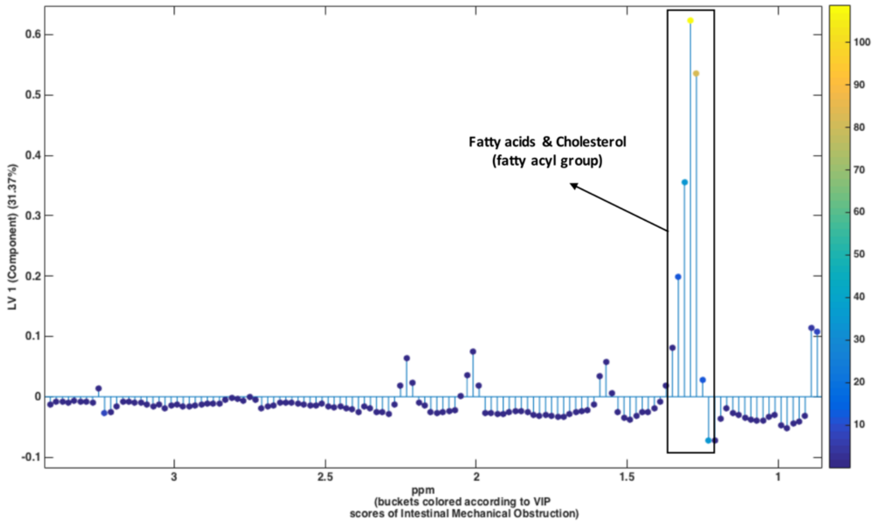

Figure S21. Loading plots of the OPLS-DA analysis from NOESY spectra focused on the variables-NMR buckets (metabolites) that contribute to the diseases discrimination. **a)** The case of intestinal ischemia. **b)** The case of intestinal strangulated obstruction. **c)** The case of intestinal mechanical obstruction. .

a

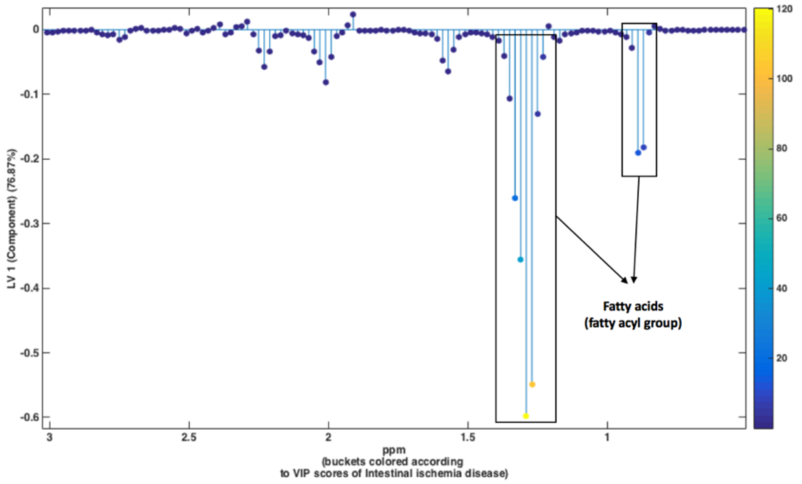

b

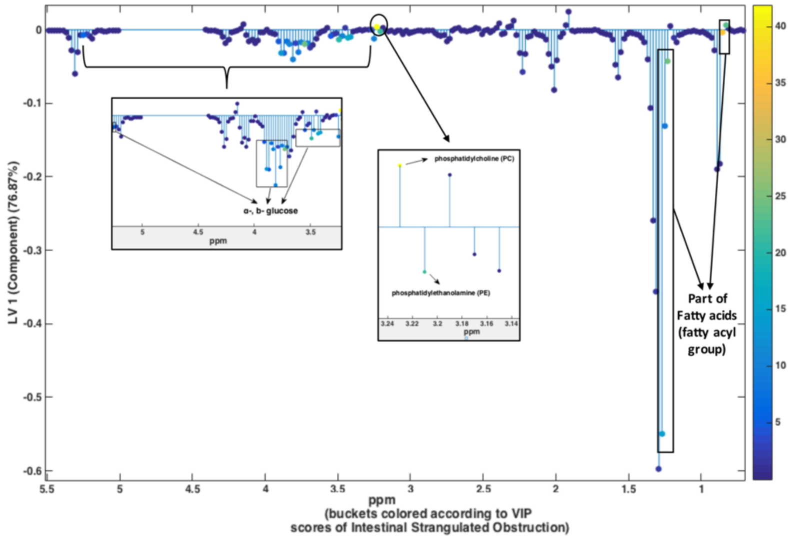

c

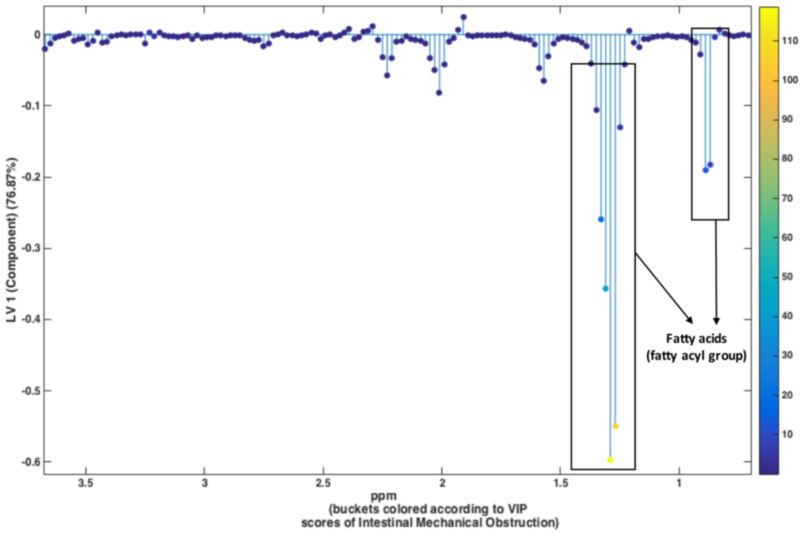

Figure S22. Loading plots of the OPLS-DA analysis from CPMG spectra focused on the variables-NMR buckets (metabolites) that contribute to the diseases discrimination. **a)** The case of intestinal ischemia. **b)** The case of intestinal strangulated obstruction. **c)** The case of intestinal mechanical obstruction. .

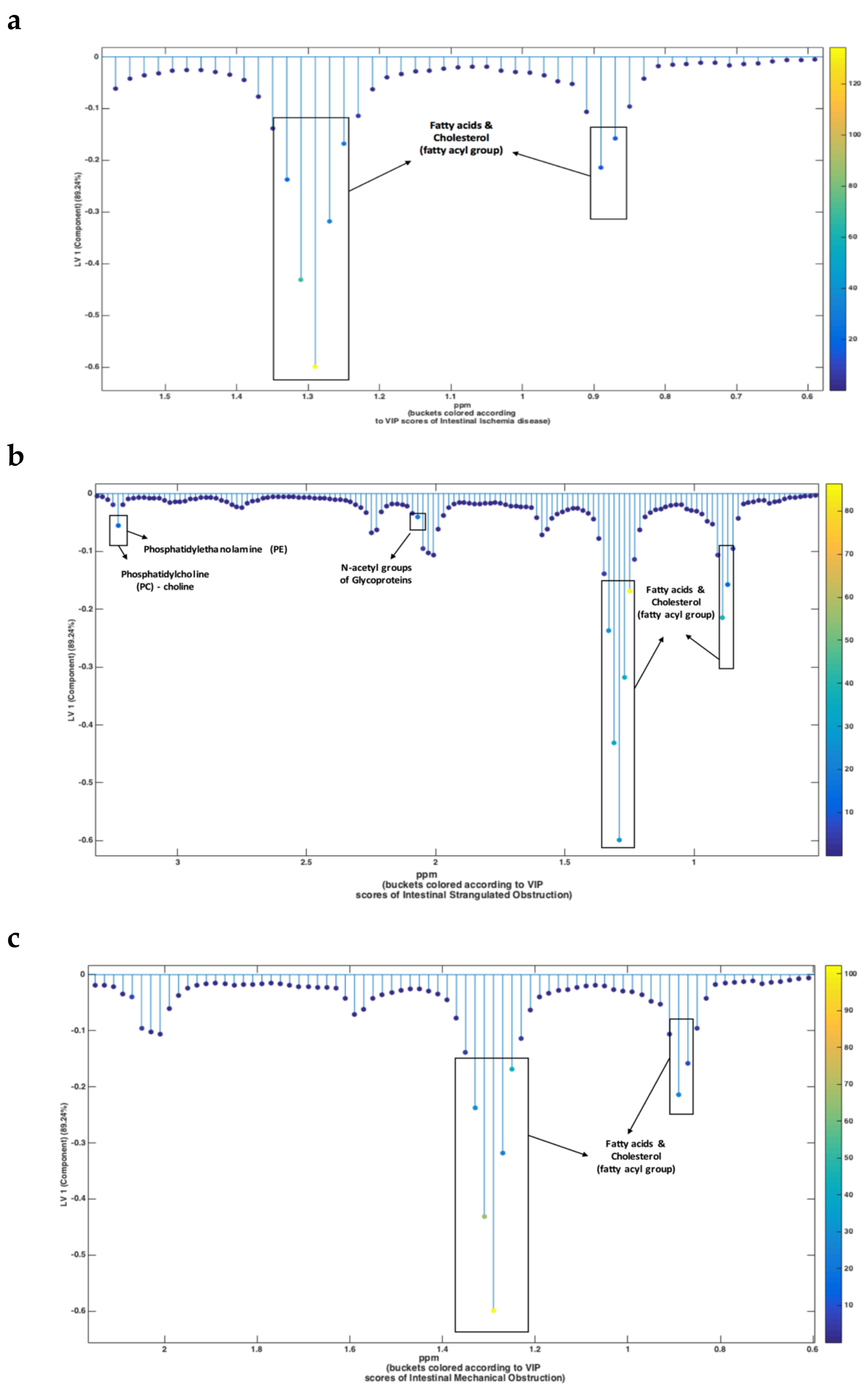

Figure S23. Loading plots of the OPLS-DA analysis from diffusion spectra focused on the variables-NMR buckets (metabolites) that contribute to the diseases discrimination. **a)** The case of intestinal ischemia. **b)** The case of intestinal strangulated obstruction. **c)** The case of intestinal mechanical obstruction. .

Table S6. Prediction probabilities of test data by the use of the CPMG model, for the intestinal ischemia, intestinal strangulated and mechanical obstruction diseases. Samples with red font are erroneously predicted, and the bold highlighted values correspond to the prediction probabilities of test data for the disease that should be assigned according to medical doctors' diagnosis.

		**CPMG model**		
**Test Samples**	**Medical doctors diagnosis for each data sample**	**Intestinal Ischemia Prediction Probability (%)**	**Intestinal Strangulated Obstruction Prediction Probability (%)**	**Intestinal Mechanical Obstruction Prediction Probability (%)**
**1**	Intestinal Ischemia	**100.0**	88.6	0.0
**2**	Intestinal Ischemia	**97.2**	0.0	0.1
**3**	Intestinal Mechanical Obstruction	0.0	0.0	**100.0**
**4**	Intestinal Mechanical Obstruction	0.0	1.8	**99.9**
**5**	Intestinal Mechanical Obstruction	79.7	0.0	**15.6**
**6**	Intestinal Strangulated Obstruction	54.4	**89.1**	1.0
**7**	Intestinal Mechanical Obstruction	95.7	0.0	**2.0**
**8**	Intestinal Strangulated Obstruction	1.0	**98.7**	18.1
**9**	Intestinal Mechanical Obstruction	1.0	0.0	**99.8**
**10**	Intestinal Ischemia	**100.0**	78.0	0.0
**11**	Intestinal Mechanical Obstruction	1.5	0.0	**97.8**
**12**	Intestinal Mechanical Obstruction	14.7	0.0	**66.5**

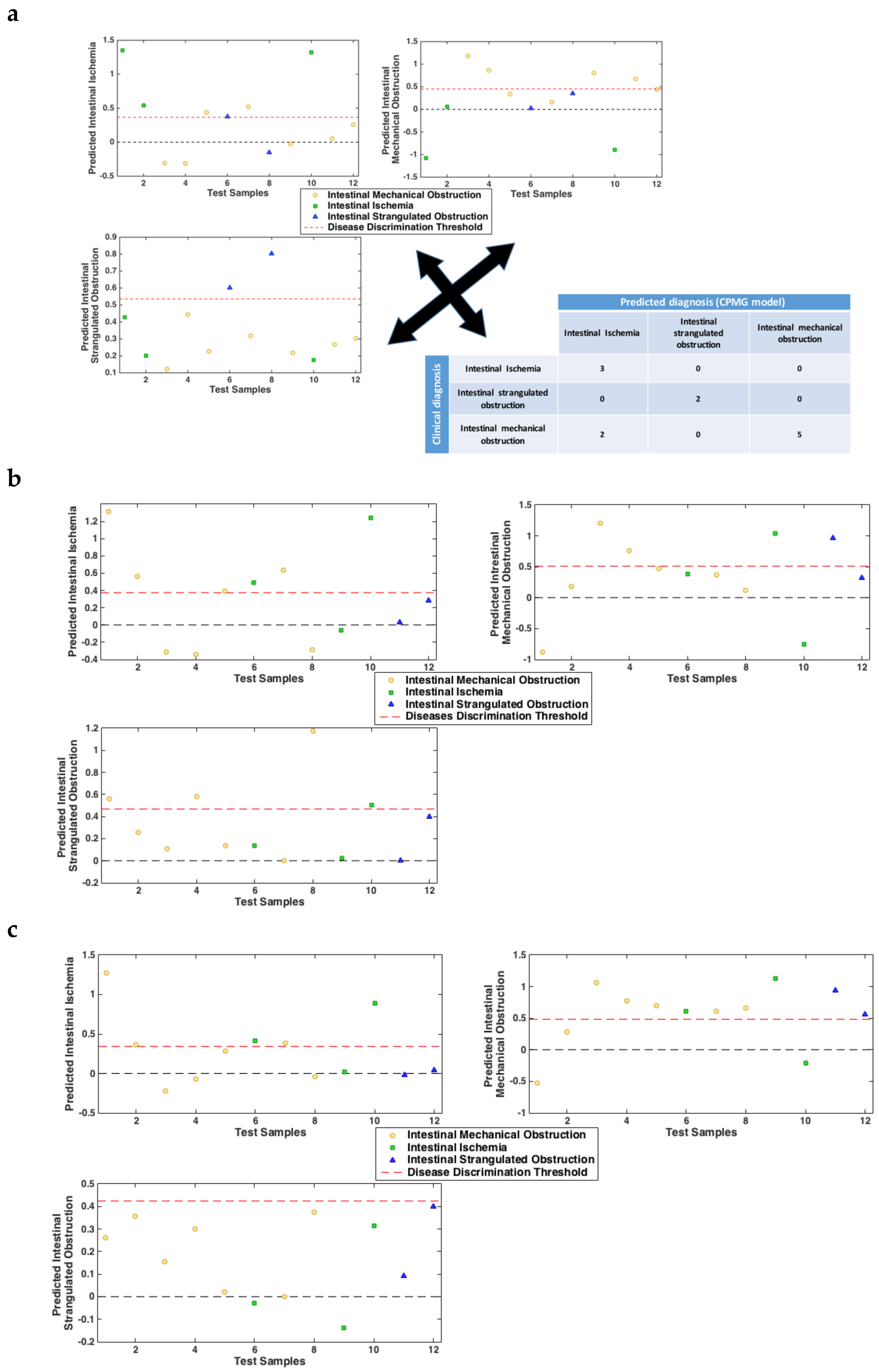

Clinical diagnosis	Predicted diagnosis (CPMG model): Intestinal Ischemia	Intestinal strangulated obstruction	Intestinal mechanical obstruction
Intestinal Ischemia	3	0	0
Intestinal strangulated obstruction	0	2	0
Intestinal mechanical obstruction	2	0	5

Figure S24. **a)** CPMG, **b)** NOESY and **c)** diffusion models prediction plots for the test data used. Above dashed red line (class discrimination threshold) is considered a successful prediction for each test data sample.
